# Atomistic Model for Water Adsorption in Mg-MOF-74: Quantum Chemical Prediction of Structures and Isotherms

**DOI:** 10.1021/jacs.6c01686

**Published:** 2026-03-16

**Authors:** Nicole Mancini, Fabian Berger, Marcin Rybicki, Kaido Sillar, Joachim Sauer

**Affiliations:** † Institut für Chemie, Humboldt-Universität zu Berlin, Unter den Linden 6, Berlin 10117, Germany; ‡ 37546 University of Tartu, Institute of Chemistry, Ravila 14a, Tartu 50411, Estonia

## Abstract

The design of improved metal–organic frameworks (MOFs) for water harvesting requires the reliable prediction of adsorption isotherms, i.e., Gibbs free energies of adsorption, with no other input than the atomic positions. We employ density functional theory (DFT) and show that, in Mg-MOF-74, well-defined adsorption structures exist for water loadings of *n* = 1, 2, 3, 4, and 5 molecules per Mg^2+^ ion. The first water molecule attaches to the open metal site, while on adsorption of subsequent molecules, structures with an increasing number of hydrogen bonds per molecule form: dimers (*n* = 2), chains in pore direction (*n* = 3), and a monolayer on the pore wall (*n* = 4). For *n* = 5, a tube-like stack of water trimers connected to the monolayer fills the pore completely, and all water molecules are 4-fold coordinated. For isotherm predictions, we use a *Multisite Langmuir* model with Gibbs free energies of −33, −19, −13, −10, and −21 kJ/mol for the steps leading to adsorption states with *n* = 1, 2, 3, 4, and 5 molecules, respectively. The close agreement of the predicted total isotherm with experiment corresponds to an accuracy of ±2 kJ/mol for Gibbs free energies. This is achieved only after adding “high-level” Coupled Cluster corrections (0, 3, 9, 8, and 11 kJ/mol for *n* = 1, 2, 3, 4, and 5, respectively) to the DFT energies. We show that the large variations observed between different experimental isotherms can be explained by sample imperfections or incomplete evacuation of samples before isotherm measurements.

## Graphical abstract

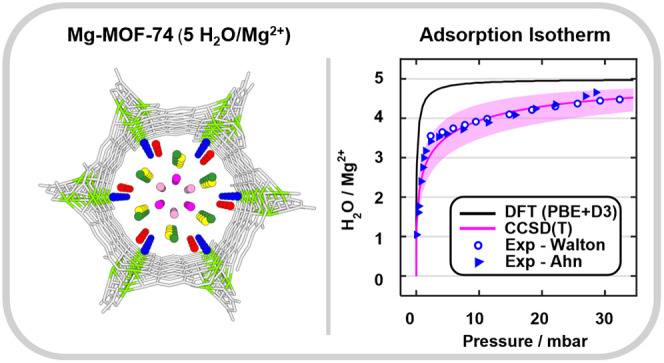

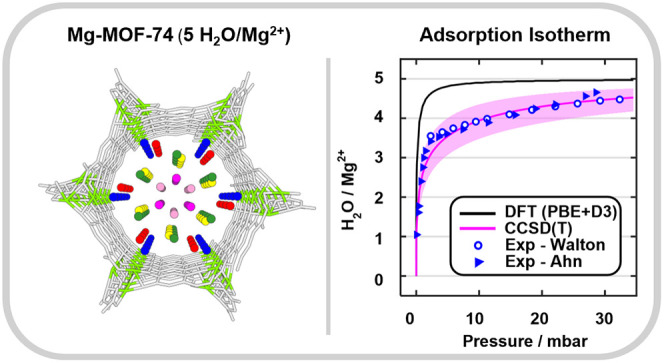

## 1 Introduction

The design of improved nanoporous materials, such as COFs (covalent organic frameworks) for carbon capture^1^ or MOFs (metal–organic frameworks) for water harvesting^2^ requires atomistic understanding of molecule–surface interactions, of the adsorption structures and the Gibbs free energy changes accompanying their formation. A diverse range of metal cations, metal oxide clusters, and functionalized organic linkers can be utilized to customize MOF structures^3–5^ for use in water harvesting devices.^6–11^ Understanding water adsorption is also of interest because water can influence gas storage and separation properties of MOFs,^12–16^ and may play a critical role in the formation and stabilization of defects.^17,18^ The key descriptor is the adsorption isotherm which represents the amount of adsorbed gas as a function of its (partial) pressure or, for water, the relative humidity at a given temperature.^2,11,19^ For MOF-303, by combination of single-crystal X-ray diffraction and density functional theory (DFT), it was possible to relate specific adsorption structures formed on increasing water loading to points of the adsorption isotherm.^8^ The insight gained has resulted in a linker extension strategy. Guided by DFT predictions, MOF-LA2-1 has been synthesized which exhibits an approximately 50% increase in water capacity compared to MOF-303.^9^

To further advance the molecular understanding of water uptake into MOF pores, we study Mg-MOF-74,^20–22^ the magnesium member of the M_2_(dobdc) isostructural series, which is a “classical” MOF used for fundamental studies.^23^ M represents a divalent metal ion and dobdc stands for 2,5-dioxido-1,4-benzenedicarboxylate. We aim to establish a model that relates the stepwise formation of distinct water structures with increasing loading to individual contributions to the adsorption isotherm. We achieve this with a local approach for isotherm predictions which involves the following steps:^24,25^ (i) DFT with periodic boundary conditions is employed to explore the potential energy surface (PES) for different loadings, i.e., to find minimum energy structures for water located at different adsorption sites. (ii) For the DFT-optimized structures, (a) Gibbs free energies of adsorption are calculated from vibrational partition functions (“analytical sampling”) and (b) Coupled Cluster theory with Single, Double, and perturbative Triple substitutions, CCSD­(T), is employed within a hybrid QM:QM scheme^24^ (QM—quantum mechanics) to achieve chemically accurate (±4 kJ/mol)^26^ adsorption energies. (iii) The Gibbs free energies obtained for the different loadings are then used within a multisite Langmuir model^25^ for isotherm predictions.

Figure 1 shows the hexagonal pore structure of Mg-MOF-74,^20–22^ also known as CPO-27-M.^27^ Dietzel et al. used powder X-ray diffraction (PXRD) to determine the structure of Mg-MOF-74, fully water-loaded with five molecules per Mg^2+^.^20^ The measured isotherms^28–30^ for water adsorption on Mg-MOF-74 vary widely, with maximum loadings ranging from 3.5 to 4.5 H_2_O/Mg^2+^, see Figure 2, and so do the isotherms obtained with different simulation methods.^31–33^ The lack of reproducibility of isotherms for nominally the same MOF has become a major point of concern.^34^ Different isotherms have been reported for samples prepared under different synthesis conditions.^29^ Moreover, structural defects such as missing linkers^35–37^ or other imperfections which obstruct access to some of the metal ion sites may affect the measured isotherms.^21^

**Figure 1:**
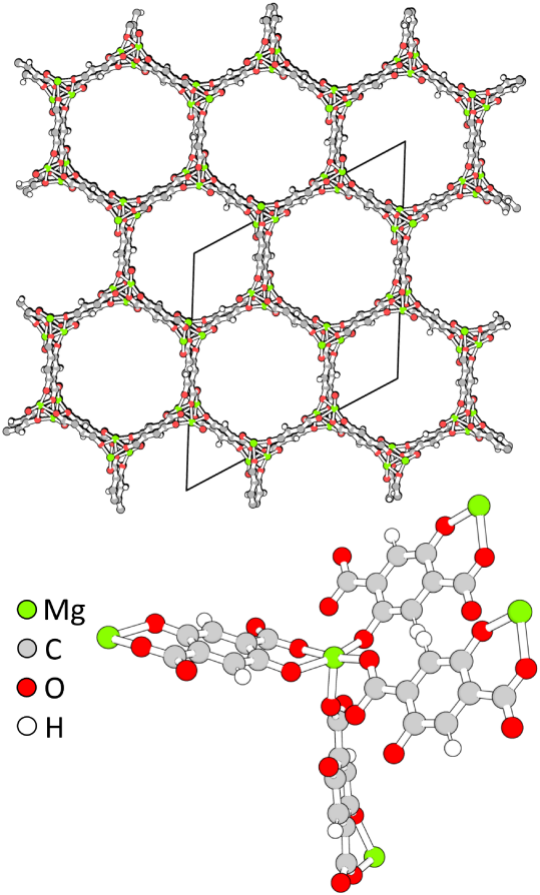
Top: view in direction of the hexagonal pores of Mg-MOF-74 with the conventional unit cell indicated in black. Bottom: coordination environment of a Mg^2+^ site.

**Figure 2:**
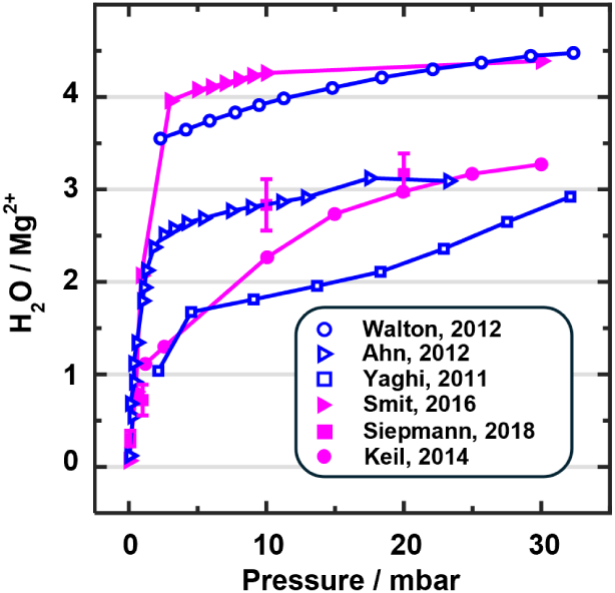
Experimental (blue) and calculated (magenta) isotherms for water adsorption in Mg-MOF-74 at *T* = 298 K. Values are taken from: Walton, 2012;^28^ Ahn, 2012;^29^ Yaghi, 2011;^30^ Smit, 2016;^31^ Keil, 2014;^33^ Siepmann, 2018 ^32^ (313 K—the expected difference to 298 K is within error bars, see Figure S5.6 in the Supporting Information).

Here, we aim to make reliable isotherm predictions for ideal MOF structures with no other input than the positions of the atoms. The wide variation of simulation results^31–33^ for the ideal Mg-MOF-74 structure shows that the methods used—all based on DFT—are not predictive. While DFT has provided atomistic understanding of adsorbate structures in MOFs,^38–41^ including water adsorption on saturated and coordinatively unsaturated metal sites,^6,8,42,43^ at the affordable level of the Generalized Gradient Approximation (GGA), DFT rarely achieves the accuracy needed for reliable predictions of adsorption energies, even if dispersion is taken into account, see e.g., ref 42. The Perdew–Burke–Ernzerhof (PBE)^44^ functional belongs to the GGA-type functionals, and, augmented with Grimme’s D3 dispersion term,^45^ was employed by Siepmann and coworkers. Also the vdW-DF2 functional used by Smit and coworkers^32^ for force field parametrization falls into this category. With our *hybrid CCSD­(T):DFT* method we go beyond DFT and reach the required accuracy of ±1–2 kJ/mol.^46^

Our PBE+D3 structure optimizations show that well-defined adsorption structures exist for water loadings of 1, 2, 3, 4, and 5 molecules per Mg^2+^ ion. We will demonstrate that the isotherms predicted with Coupled Cluster (CC)-level Gibbs free energies are in close agreement with those measured isotherms^28,29^ that reach a close-to-maximum loading of 4.5 H_2_O/Mg^2+^ at about 30 mbar. We will conclude that near-perfect samples have been used in those measurements. We will further show that blocked metal sites or incomplete evacuation of the pores prior to measurement provide an explanation for the variations between the isotherms measured by different researchers.^2,28–30^

## 2 Models and Methods

### 2.1 Periodic MOF Models

The conventional unit cell (CUC) of Mg-MOF-74 is obtained from the Cambridge Crystal Database (CSD number 668974).^20^ Starting from the experimental structure,^20^ protons are added to the benzene ring of the (dobdc)^4–^ linker. The primitive unit cell is transformed into a Niggli reduced cell, which is used for all calculations with periodic boundary conditions, for details see the Supporting Information (SI), Section S1.

### 2.2 Methods for Gibbs Free Energies of Adsorption

Predictions of adsorption constants, i.e., Gibbs free energies of adsorption, with “chemical” accuracy (±4 kJ/mol)^26^ that is comparable to experiment, for realistic models with hundreds of atoms in the simulation cell, as we study here, is a challenging problem of computational quantum chemistry. We use an ab initio divide-and-conquer approach for free energy predictions^24,25,46,47^ that (i) combines a high-level QM description of the adsorption site with a low-level QM description of the full periodic solid to calculate energies,^24,48,49^ and (ii) samples the PES locally with vibrational partition functions (“analytical sampling”). At the low level, we apply DFT and employ the Perdew–Burke–Ernzerhof functional with Grimme’s D3 dispersion augmentation,^44,45^ PBE+D3, for structure optimizations and harmonic force constant calculations. The Vienna Ab Initio Simulation Package (VASP) version 5.3.5,^50,51^ is applied, which uses periodic boundary conditions and employs plane wave basis sets for valence electrons and the projector augmented wave (PAW) method for core electrons. For cluster models C, high-level corrections, ΔCC, are calculated at the PBE+D3 structures as difference between the CCSD­(T) and PBE+D3 energies, Δ*E* ^CCSD(T)^(C) and Δ*E* ^PBE+D3^(C), respectively:

$$\Delta \mathrm{CC}\left(C\right)=\Delta E^{\mathrm{CCSD}(T)\;}\left(C\right)-\Delta E^{\mathrm{PBE}+D3\;}\left(C\right)$$ 1

CCSD­(T) calculations for all pairwise H-bond interactions between water molecules and clusters cutout of the MOF framework, as well as between water molecules. Tests have shown that three- and four-body contributions to ΔCC are negligible, see Section S2.2 of the SI. The domain-based local pair natural orbital (DLPNO) method^52^ as implemented in ORCA, version 4.2.1 ^53,54^ is employed, see Section S2.2 in the SI for further details.

Adding the high-level correction to the PBE+D3 energy of the full periodic structure, Δ*E* ^PBE+D3^(S), defines an approximation, Δ*E* ^CCSD(T):PBE+D3^(C,S), for the CCSD­(T) adsorption energy of the periodic system, Δ*E* ^CCSD(T)^(S):

$$\Delta E^{\mathrm{CCSD}(T)\;}\left(S\right)\approx \Delta E^{\mathrm{CCSD}\left(T\right):\mathrm{PBE}+D3\;}\left(C,S\right)=\Delta E^{\mathrm{PBE}+D3\;}\left(S\right)+\Delta \mathrm{CC}\;\left(C\right)$$ 2

For simplicity, we will refer to these *hybrid CCSD­(T):PBE+D3* results as “CCSD­(T)” or simply as “CC” results hereafter.

The average adsorption energy for *n* water molecules per Mg^2+^ site, Δ*E_n_*, is obtained from the total energy of the MOF loaded with *n* water molecules, $E_{nH_{2}O/\mathrm{MOF}}$ , the total energy of the bare MOF, *E*_MOF_, and the energy of *n* isolated water molecules in the gas phase, $E_{H_{2}O\left(g\right)}$ :

3 $$\Delta E_{n}=\frac{1}{n}\left(E_{nH_{2}O/\mathrm{MOF}}-n\cdot E_{H_{2}O\left(g\right)}-E_{\mathrm{MOF}}\right)$$

The corresponding adsorption enthalpies are obtained as

$$\Delta H_{n}=\Delta E_{n}+\Delta E_{\mathrm{ZPV}}+\Delta E_{\mathrm{therm}}-RT$$ 4

The Gibbs free energy of adsorption per adsorbed molecule, is defined as the adsorption enthalpy, Δ*H*_*n*_, minus the entropy term, *T*Δ*S*_*n*_:^55^

5 $$\Delta G_{n}=\Delta H_{n}-T\Delta S_{n}$$

The zero-point vibrational energy, Δ*E*_ZPV_, as well as thermal energy contributions, Δ*E*_therm_, and adsorption entropies, Δ*S*_*n*_, at a given temperature *T*, are calculated from harmonic vibrational partition functions^55^ obtained with PBE+D3, and R is the ideal gas constant.

For each consecutive adsorption step, *n* = 1–5,

6 $${\left(H_{2}O\right)}_{n-1}/\mathrm{MOF}+H_{2}O_{\mathrm{gas}}⇄{\left(H_{2}O\right)}_{n}/\mathrm{MOF}$$

the Gibbs free energy for adsorption of the *n*-th water molecule is defined as

7 $$\Delta G_{n-1\to n}=nG_{n}-\left(n-1\right)G_{n-1}-G_{H_{2}O}$$

*G_n_* and *G_n_*_–1_ are the Gibbs free energies per water molecule for loadings *n* and *n –* 1, respectively, and $G_{H_{2}O}$ is the Gibbs free energy of a water molecule in the gas-phase.

The Gibbs free energies are used as input for model isotherms.^25,46,47^ We adopt a *Multisite Langmuir* model for our isotherm predictions, details are given below.

### 2.3 Multisite Langmuir Model with Independent Sites

Our local approach to free energy calculations uses model isotherms to relate Gibbs free energy changes to isotherms, i.e., to calculate the adsorbed amounts at a given pressure of the adsorbed gas.^25,46,47^ We assume simultaneous availability of all sites for water adsorption, including the empty MOF as well as sites A, AB, ABC, and ABCD:

$$0+H_{2}O\overset{K_{1}}{\to }A$$ 8a

$$A+H_{2}O\overset{K_{2}}{\to }AB$$ 8b

$$AB+H_{2}O\overset{K_{3}}{\to }ABC$$ 8c

As water molecules are added, the population of the five states A, AB, ..., ABCDE is determined from the Gibbs free energy changes for forming these states at a given temperature, see eq 7, under the assumption that these states are independent.

The equilibrium adsorption constant for formation of loading *n* is:

9 $$K_{n}=\exp\left(-\frac{\Delta G_{n-1\to n}}{RT}\right)$$

The coverage for each of the sites with *n* water molecules per Mg^2+^ at pressure *P* is given by the Langmuir isotherm:

10 $$\theta_{n}=\frac{K_{n}P}{1+K_{n}P}$$

The total coverage at a given pressure and temperature is the sum of the coverages of all five sites:

11 $$\theta_{\mathrm{total}}=\overset{5}{\underset{n=1}{\sum }}\theta_{n}$$

## 3 Results and Discussion

### 3.1 Search for Adsorption Structures

To explore the PES for water molecules in the pores of Mg-MOF-74 and to identify the energetically most stable adsorption structures, we started optimizations from various initial arrangements of water molecules. For the stationary points, we calculated the Hessians. When negative eigenvalues were detected, small displacements along the corresponding normal modes were made to search for more stable configurations in nearby regions of the PES. This approach ensures broad sampling of relevant adsorption structures. We also performed molecular dynamics (MD) simulations to generate additional starting structures for optimizations. For all loadings, symmetric structures keeping the *R*3̅ space group of the MOF framework were found to be the most stable. Energies and enthalpies of adsorption as well as imaginary wavenumbers, if any, for all stationary points localized are listed in Table S3.6 in the SI.

The obvious adsorption sites for the first water molecules up to a loading of 1 H_2_O/Mg^2+^ are the Mg^2+^ ions,^43^ where they complete the (6-fold) coordination sphere of the Mg^2+^ ions. The water molecules saturate the “open metal” or “coordinatively unsaturated metal” sites of this MOF,^56^ see Figure 1. For a single water molecule (loading ^1^/_6_ H_2_O/Mg^2+^) binding is 1 kJ/mol stronger than for occupation of all six Mg^2+^ sites, −81.2 compared to −80.2 kJ/mol. Thonhauser and coworkers^43^ applied the van der Waals-density functional (vdW-DF) and obtained −76 compared to −73 kJ/mol for ^1^/_6_ compared to 1 H_2_O/Mg^2+^. For 1 H_2_O/Mg^2+^, we have also considered three water dimers attached to one Mg^2+^ site each, and two water trimers attached to two Mg^2+^ sites each, see Figure 3. They both are less stable than the symmetric distribution over all six Mg^2+^ sites, which we label A-sites.

**Figure 3:**
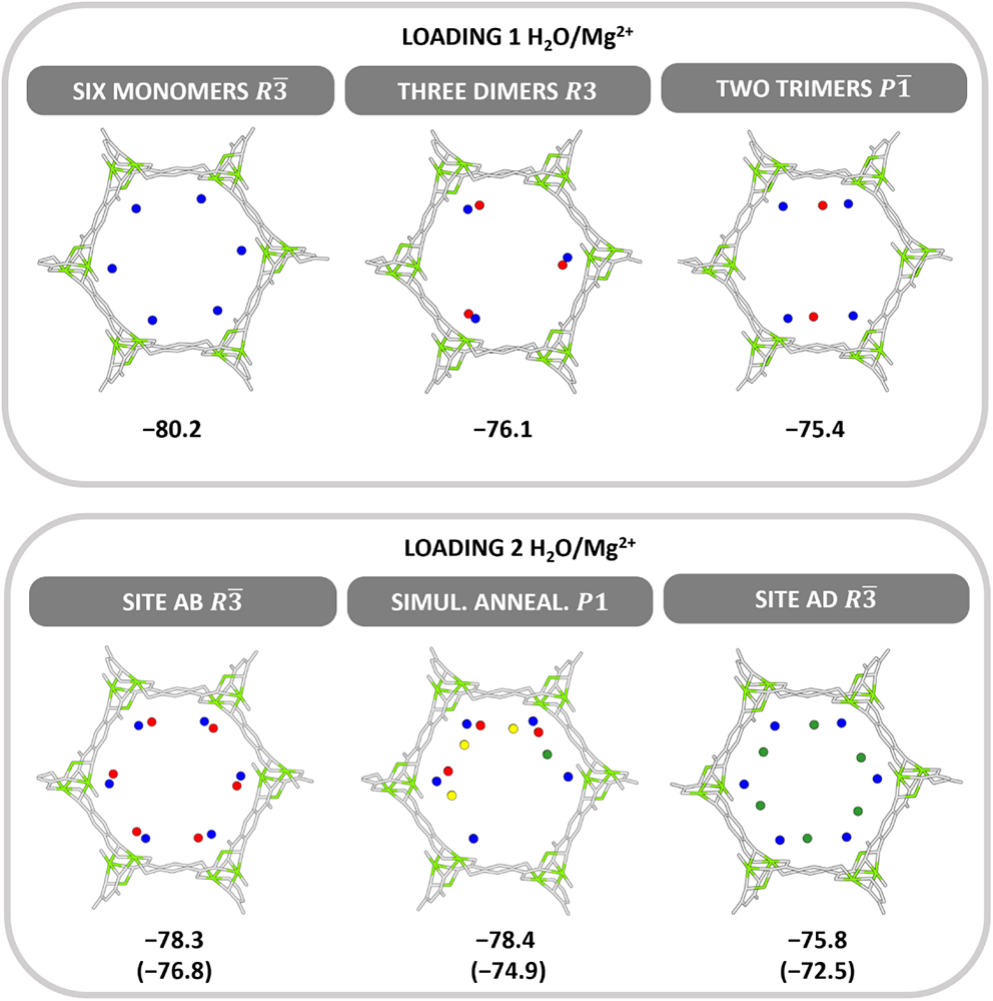
Different distributions of six water molecules over the six Mg^2+^ sites for 1 H_2_O/Mg^2+^ (top) and different adsorption structures for 2 H_2_O/Mg^2+^ (bottom). The space group of all structures is given as well as their PBE+D3 adsorption energies in kJ/mol. In parentheses: (PBE+D3)+ΔCC results.

For a loading of 2 H_2_O/Mg^2+^, the symmetric **AB** structure with water dimers attached to each of the six Mg^2+^ ions is themost stable, see Figure 3. The second water molecule (**B** site) accepts a hydrogen bond (H-bond) from the molecule at the Mg^2+^ ions (**A** site) and donates one to a phenolic O atom of the framework, see Figure S3.3 in the SI. MD simulations with simulated annealing resulted in a nonsymmetric structure also shown in Figure 3. One can distinguish three water dimers (with H-bonds to additional water molecules colored yellow), a single water molecule located in an **A** site, and one empty Mg^2+^ site. The adsorption energy agrees with that of the **AB** structure within 0.1 kJ/mol, but when adding the Coupled Cluster correction, ΔCC, the symmetric **AB** structure becomes 2 kJ/mol more stable. The water dimers can also approach the Mg^2+^ ions in a different orientation such that the second water molecule donates an H-bond to the carboxylate O atom of the framework. This is shown in Section S3.1.3 of the SI. For reasons given below we name this site **D.** The **AD** dimers, shown in Figure 3, are 4.3 kJ/mol less stable than the **AB** dimers.

For the third water molecule per Mg^2+^ ion, we considered a bridging position (**C**) between the **AB** dimers which creates a H-bonded −B–A–C–B–A– chain in pore direction, see Figures 4 and 5. The average number of three H-bonds per molecule (Table 1) explains why this is the most stable structure among the ones considered for loading 3. Placing the third water molecule in position **D** instead of **C** yields several stationary points, but no minimum. An alternative structure with water molecules located in **ADC** sites is 16.5 kJ/mol less stable than the **ABC** structure.

**Figure 4:**
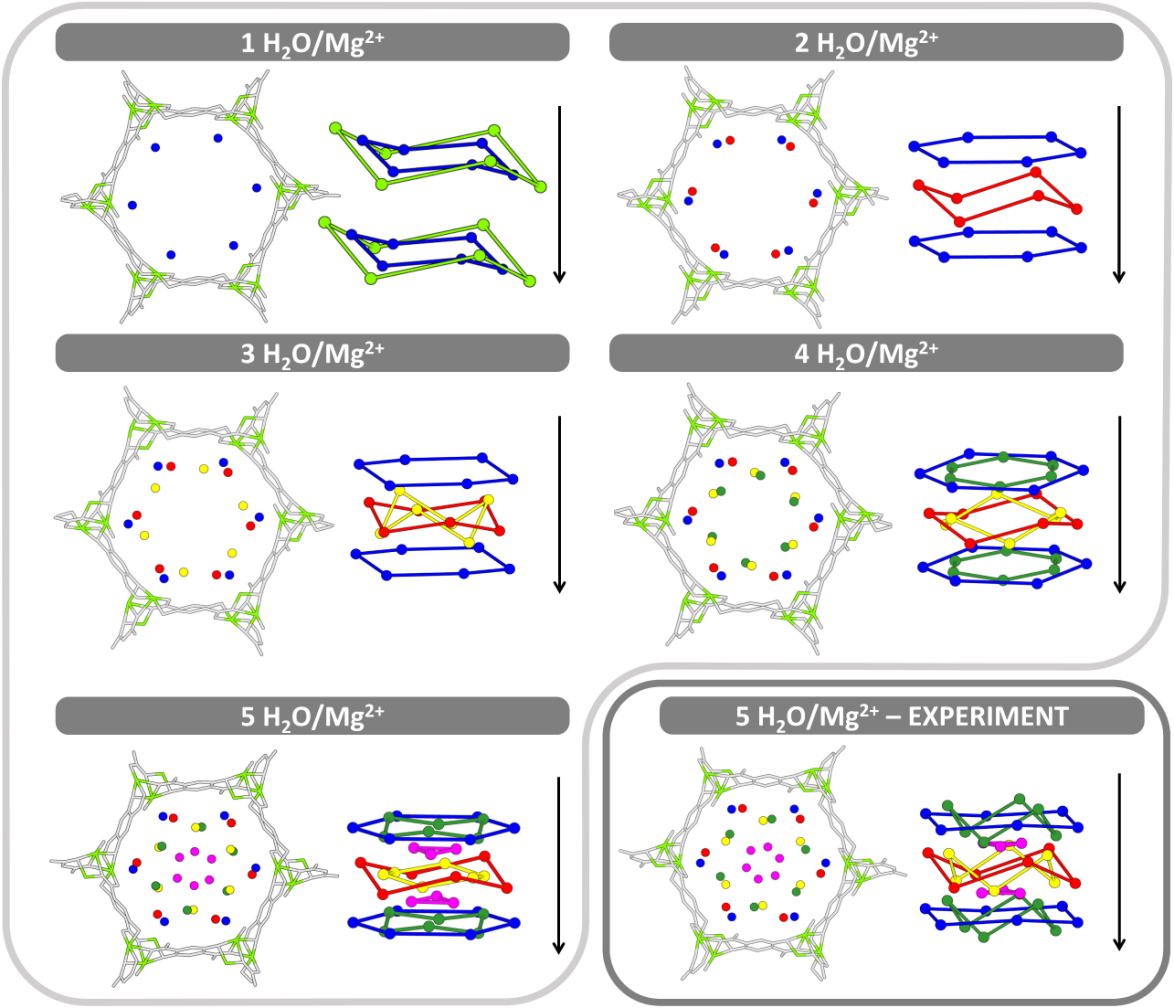
DFT (PBE+D3) optimized adsorption structures for 1 to 5 H_2_O molecules per Mg^2+^ in Mg-MOF-74, Mg_2_(dobdc)·(H_2_O)_2*n*_ with *n* = 1 to 5 (space group *R*3̅). The empty “open” Mg^2+^ site is colored light green, and the (dobdc)^2^ linker gray. Color code for adsorption sites: A—blue, B—red, C—yellow, D—dark green, and E—magenta. Bottom right panel: PXRD structure of Mg-MOF-74 fully loaded with 5 H_2_O molecules per Mg^2+^ (space group *R*3̅).^20^ Only the oxygen atoms of the water molecules are shown. Left: view into pore direction (projection on ab plane). Right: stacking in pore direction (*c*-axis, arrow). Symmetry equivalent sites are connected by lines for visual guidance. The lines do not indicate the H-bond network.

**Figure 5:**
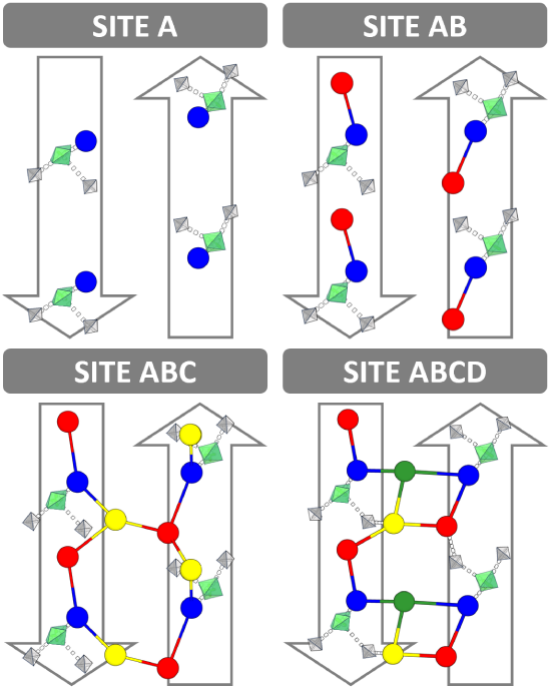
Network of H-bonds between water molecules in different adsorption sites of Mg-MOF-74 for different loadings: site A—blue, site B—red, site C—yellow, site D—dark green. Connecting lines indicate H-bonds. Selected framework sites are shown as green octahedra (Mg) and gray tetrahedra (phenolic and carboxylate O-acceptor linker sites). The view is onto the pore wall. One third of the wall is shown. The up and down arrows pointing in pore direction reflect the S_6_ symmetry.

**Table 1:** Number of Donor (D) and Acceptor (A) Bonds of Water Molecules in Sites A–E for Loadings *n* = 2–5 H_2_O/Mg^2+^ ^a^,^d^

	*n* = 2			*n* = 3		*n* = 4		*n* = 5	
Site		*D*, *A*			*D*, *A*		*D*, *A*		*D*, *A*
A^b^	Mg → A	1, 1		Mg → A	2, 1	Mg → A	2, 2	Mg → A	2, 2
	A → B			A → B		A → B		A → B	
				A → C		A → D		A → D	
B	B → O_Ph_	1, 1		B → O_Ph_	2, 2	B → O_Ph_	2, 2	B → O_Ph_	2, 2
				B → C		B → C		B → C	
C				C → B	1, 2	C → B	2, 2	C → B	2, 2
						C → O_Ox_		C → E	
D						D → A	2, 1	D → A	2, 2
						D → C		D → C	
E								E → D	2, 2
								E → E	
*m* ^c^	4/2 = 2 (1.5)			10/3 = 3.33 (3)		15/4 = 3.75 (3.5)		20/5 = 4 (3.8)	

a Ph and Ox denote phenolic and carboxylate O-acceptor sites, respectively, at (dobdc)^4–^ linkers.

b Water molecules in sites **A** change the coordination of the Mg^2+^ ions from 5 to 6, they are “saturating the open metal sites”.

c In parentheses: average number of H-bonds.

d The average coordination number of a water molecule is *m.*

Since the **A**, **B**, and **C** sites are also present in the experimental fully water-loaded structure (5 H_2_O/Mg^2+^), we removed the inner layer oxygen atoms from the experimental structure to prepare starting structures for loading 4 H_2_O/Mg^2+^. Since only the positions of the oxygen atoms are available from experiment, the H-bond pattern was unknown and several patterns were considered. Optimizations resulted in stationary points, but only in one case a local minimum was found. In this **ABCD** structure the fourth molecule is in the same position as the second molecule in the less stable **AD** structure for loading 2. For loading 5 the O atom positions were taken from the experimental structure and three different models were considered for the H atom positions. Optimizations resulted in stationary points, but the **ABCDE** structure was the only minimum found after distortions along modes with imaginary wavenumbers.

### 3.2 Final Adsorption Structures

With increasing loading, water molecules populate distinct adsorption sites in the pores of Mg-MOF-74, denoted as **A**, **B**, **C**, **D**, and **E**, see Figure 4. Table 1 summarizes the H-bonds formed with MOF surface sites and between water molecules at different loadings. Further structure details, i.e., distances and angles obtained by PBE+D3 structure optimization, are provided in the SI, Section S3. H-bonds are defined based on distance and angle criteria. For O–O distances, we consider a range from 264 to 365 pm, and for O–H···O angles a range from 150° to 176°, consistent with commonly used H-bond definitions.^57,58^

Building on the description of the water molecule positions, we now focus on the H-bond network. The first water molecules (1 H_2_O/Mg^2+^) attach to the Mg^2+^ ions (**A** site), where they complete the (6-fold) coordination sphere of the Mg^2+^ ions. The second water molecule (**B** site, 2 H_2_O/Mg^2+^) accepts an H-bond from the molecule at site **A**. It is particularly strong because the protons of the first water molecule are more acidic due to the interaction with the Mg^2+^ cation. The **A**–**B** water dimer is further stabilized by an additional H-bond between the molecule at site **B** and a phenolic oxygen (O_Ph_) of the (dobdc)^4–^ linker. The third water molecule (**C** site, 3 H_2_O/Mg^2+^) bridges the separated **A**–**B** dimers, creating a H-bonded −**B**–**A**–**C**–**B**–**A**– chain in pore direction, see Figure 5. The molecules at site **C** interconnect these chains perpendicular to the pore direction by forming a third H-bond with a water molecule at site **B** of the neighboring chain. Adding a fourth molecule per Mg^2+^ site (site **D**, 4 H_2_O/Mg^2+^) completes a monolayer on the internal MOF surface. Site **D** is located between **C** and **A** in pore direction, forming −**B**–**A**–**D**–**C**– chains, see Figure 5. The latter are interconnected with **A**–**D** bonds forming −**A**–**D**–**A**–**D**– chains perpendicular to the pore direction. This completes a monolayer of water molecules, in which three of the four molecules per Mg^2+^ have the maximum coordination of four, only the molecules in **D** sites are stabilized by three H-bonds. This results in an average coordination number of 3.75. The fifth water molecule per Mg^2+^ locates on-top of the **ABCD** monolayer (site **E**) and completely fills the MOF pores. The molecules at the **E** sites form a tube-like stack of water trimers in pore direction with *R*3̅ symmetry (Figure 6). Each molecule of the trimers accepts an H-bond from molecules in **C** sites and donates an H-bond to molecules in **D** sites. This results in the maximum coordination number of four for all water molecules.

**Figure 6:**
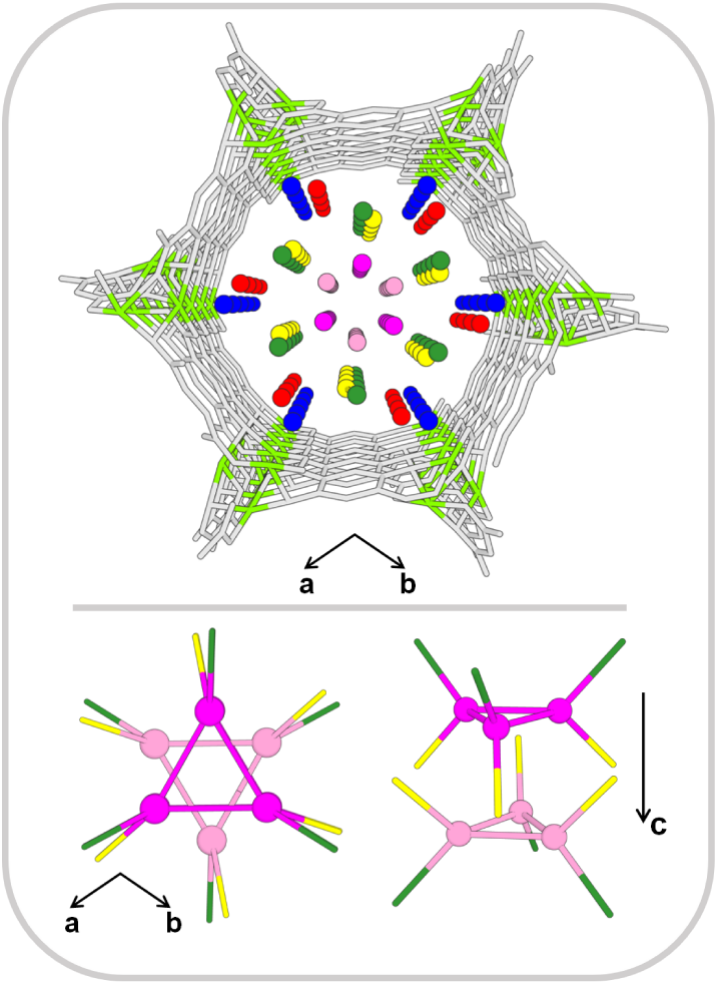
Adsorption structure of fully water-loaded Mg-MOF-74 with 5 H_2_O/Mg^2+^. Top: view into the hexagonal pore. Bottom: two different views of the stacked water trimers at **E** sites (magenta and pink). They form a tube in pore direction and are connected to the **ABCD** monolayer by H-bonds to molecules at sites **C** (yellow) and **D** (green).

Differently from water adsorption in MOF-303, for which water loading has been followed by single-crystal XRD measurements,^9^ for Mg-MOF-74 a powder XRD structure^20^ is available only for the fully water-loaded MOF with five water molecules per Mg^2+^ ion, Mg_2_(dobdc)·(H_2_O)_10_. Figure 4 provides a comparison of the oxygen positions of our PBE+D3 optimized structure (space group *R*3̅) with the experimental powder XRD structure.^20^ The overall arrangement of the water molecules is the same, but PBE+D3 tends to underestimate the distances between water molecules, see e.g., refs 59,60 . This results in more planar water arrangements compared to the experimentally observed, more undulated configurations. The predicted O···O distances between water molecules at sites **A**, **B**, **C**, **D**, and **E** differ from experimental values by 2 to 68 pm. The O–O–O angles formed by water molecules at the three consecutive symmetry-equivalent sites (**A**, **B**, and **E**) show only minor deviations (smaller than 4.5°) for O_A_, O_B_ and O_E_, while more pronounced deviations of 20° and 22° are observed for the O_C_–O_C_–O_C_ and O_D_–O_D_–O_D_ angles, respectively.

### 3.3 Adsorption Energies and Thermodynamic Functions

Table 2 shows the energies and different thermodynamic quantities for each consecutive adsorption step, *n* – 1 → *n*, see eq 7. The corresponding quantities for loadings *n* = 1–5 as defined in eqs 3 to 5 are given in Section S4.1 of the SI. The adsorption enthalpies are primarily governed by the number and nature of intermolecular bonds at each loading. The strong interaction between Mg^2+^ ions and water molecules results in a PBE+D3 adsorption enthalpy of −74 kJ/mol for the first step, 0 → **A**. Formation of H-bonds in the steps leading to **AB** dimers, connected **ABC** chains, and **ABCD** monolayers is less exothermic with Δ*H* values between −66 and −69 kJ/mol. The completion of a three-dimensional network in the **ABCDE** tube with 4-fold coordination for each molecule is most exothermic, −82 kJ/mol. Changes of the zero-point vibrational energies contribute as much as 8 to 15 kJ/mol, up to 18%, to the predicted heat changes which indicates that quantum effects of nuclear motion cannot be ignored when calculating thermodynamic functions of adsorption.

**Table 2:** Electronic Energies, Δ*E*, Zero Vibrational Point Energies, Δ*E*_ZPV_, Thermal Energy Contributions, Δ*E*_therm_, Standard (298 K and 0.1 MPa) Enthalpies, Δ*H*, Entropy Contributions, −*T*Δ*S*, and Gibbs Free Energies, Δ*G*, for the Five *n* – 1 → *n* Adsorption Steps, All in kJ/mol^a^ ^,^ ^b^

	PBE+D3						CCSD(T)		
Step	Δ*E*	Δ*E*_ZPV_	Δ*E*_therm_	Δ*H*	–*T*Δ*S*	Δ*G*	ΔCC	Δ*H+*ΔCC	Δ*G+*ΔCC
0 → A	–80.1	7.9	0.5	–74.2	40.7	–33.5	0.3	–73.9	–33.2
A → AB	–76.5	12.1	–2.2	–69.0	47.1	–22.0	2.9	–66.1	–19.1
AB → ABC	–73.2	11.4	–1.5	–65.7	44.4	–21.3	8.6	–57.1	–12.7
ABC → ABCD	–77.1	14.6	–4.1	–69.1	51.2	–17.9	7.5	–61.6	–10.4
ABCD → ABCDE	–91.0	14.9	–3.1	–81.7	50.2	–31.5	10.6	–71.1	–20.8
A → AD	–71.5	11.6	–1.8	–64.3	44.1	–20.2	6.6	–57.7	–13.6

a All energies and thermodynamic functions are obtained with PBE+D3 within the harmonic approximation.

b CCSD­(T) high-level corrections to the adsorption energies, ΔCC, and their effect on the enthalpies, Δ*H*+ΔCC, and Gibbs free energies, Δ*G*+ΔCC, are reported as well.

Since PBE+D3 overestimates the stability of H-bonds,^59,61–63^ the CCSD­(T) corrections, ΔCC, increase with the average number of H-bonds from 2.9 to 10.6 kJ/mol for **AB** with 1.5 H-bonds per water molecule to **ABCDE** with 3.8 H-bonds, respectively. Whereas Δ*H* remains virtually unaffected for the first adsorption step (−74 kJ/mol), the CCSD­(T) corrected Δ*H* values for the next steps, leading to the **AB**, **ABC**, and **ABCD** H-bonded structures, become less exothermic, between −57 and −66 kJ/mol. The last adsorption step, leading to the **ABCDE** structure, remains the most exothermic, −71 kJ/mol, among the H-bonded structures, but is less exothermic than the first adsorption step in which the water molecules attach to the Mg^2+^ ions.

The entropy loss, −*T*ΔS, is smallest (41 kJ/mol) for the **A** site molecules because their movements are less restricted than those of molecules at other sites which are constrained by H-bonds. As adsorption at this site is also most exothermic, the final Δ*G*+ΔCC value is by far more negative for the first step (−33 kJ/mol) than the subsequent H-bond forming steps with Δ*G*+ΔCC values between −21 and −10 kJ/mol.

As mentioned before, we have identified a second structure, **AD**, for adsorption of the second water molecule. Since the high-level correction for **A** → **AD** is significantly more positive than for **A** → **AB**, the Gibbs free energy is 5.5 kJ/mol less exergonic. Therefore, we will not further consider formation of the **AD** structure here, for more information see Section S3 in the SI.

## 4 Comparison to Experiments

The left panels of Figure 7 compare our *Multisite Langmuir* model predictions (Gibbs free energies of adsorption from Table 2) to the measurements of Walton and coworkers^28^ and of Ahn and coworkers.^29^ For the CCSD­(T)-corrected energies, Δ*G*+ΔCC in Table 2, our prediction is in convincing agreement with both experiments.^28,29^ This implies that these two measurements used samples which closely resemble the ideal Mg-MOF-74 structure model that we adopted for the calculations, see Section 2.1. We have also tested a *Step-wise Langmuir* model, see Section S5.2 of the SI. It yields similar results, but since the simpler *Multisite model* matches the experimental isotherms more closely, we refer to the latter in the following.

**Figure 7:**
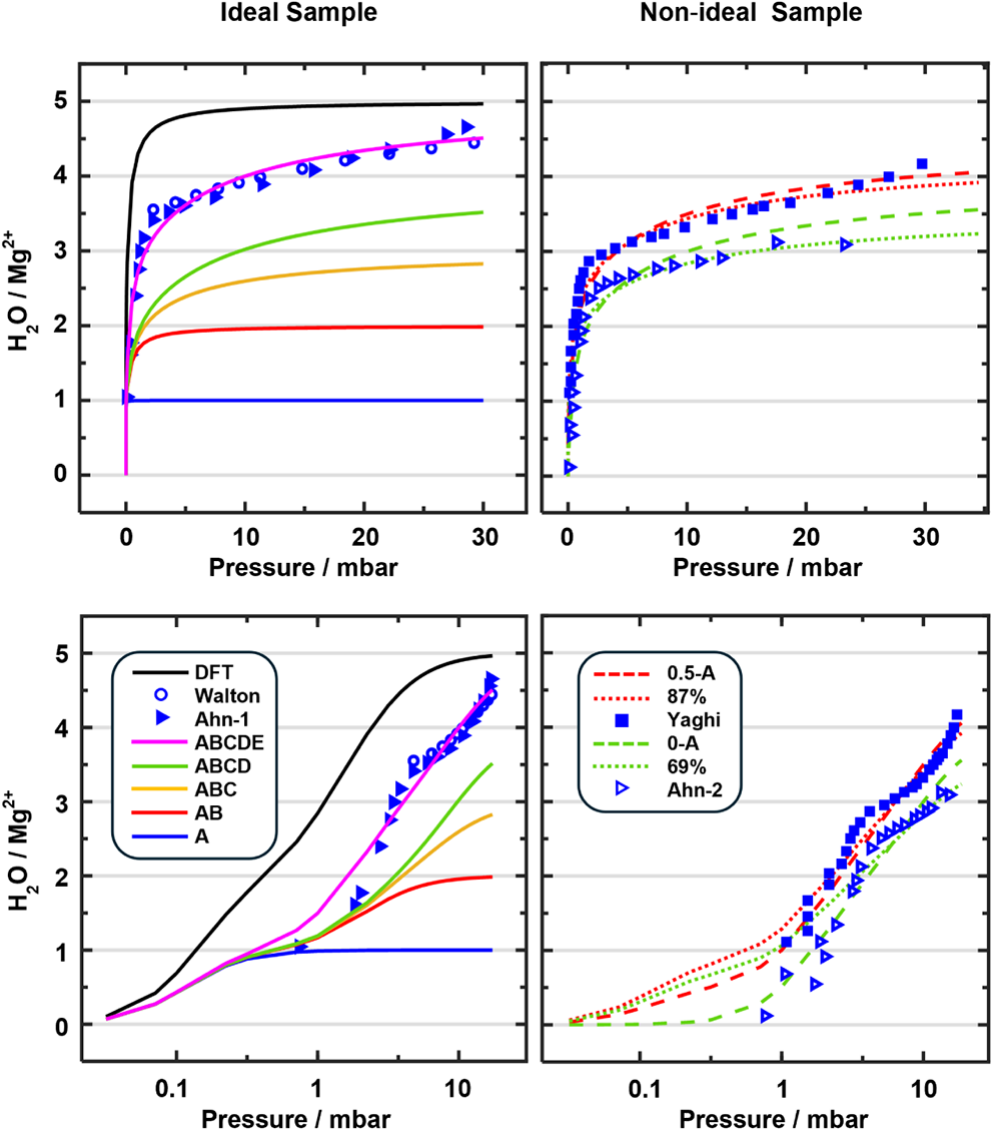
Predicted adsorption isotherms (*Multisite Langmuir* model) compared to experiment (298 K). Left: PBE+D3 (DFT, black) and CCSD­(T) results (ABCDE, magenta). In addition, CCSD­(T) results are plotted for each state, ABCD—green, ABC—yellow, AB—red, A—blue. The corresponding Gibbs free energies are reported in Table 2. Experimental isotherms (blue symbols) are taken from Walton^28^ (triangles) and Ahn^29^ (squares). Right: CCSD­(T) isotherms assume that half or all of the A sites are inactive (0.5-A and 0-A, broken red and green lines, respectively) or that only 87% or 69% of all sites are available for adsorption (dotted red and green lines, respectively). Experimental isotherms (blue symbols) are taken from Yaghi^2^ (squares) and Ahn^29^ (triangles) for a sono-chemically prepared sample (Ahn-2, triangles). Bottom: Logarithmic pressure scale.

Figure 7 shows also the individual contributions of the five adsorption sites to the total CCSD­(T) isotherm. Whereas close-to-full loading (4.5 H_2_O/Mg^2+^) is reached only at 30 mbar, all **A** sites are filled already at 1 mbar, and at 10 mbar also **B** sites are fully occupied. The level of agreement with experiment which corresponds to an accuracy of ±2 kJ/mol for the calculated Gibbs free energies, see Section S5.1 in the SI, validates our local approach with an adsorption model based on distinct structures which are filled with increasing loading. Previously, using the same local approach, it was also possible to establish an adsorption model with different sites for methane in Mg-MOF-74.^25^ As for water, adsorption is strongest at Mg^2+^ sites, followed by linker sites and second layer sites. Differently from water, however, the “lateral” interactions between the adsorbed methane molecules are weak.

With the PBE+D3 Δ*G*-value*s* in Table 2, the *Multisite Langmuir* model (black curve in Figure 7) predicts a much steeper increase of the coverage with pressure than obtained with the reference CCSD­(T) data (magenta curve) and observed in experiments for supposedly defect-free samples.^28,29^ The PBE+D3 isotherm saturates at a too low pressure. At about 30 mbar partial pressure, the water uptake is about 0.5 H_2_O/Mg^2+^ higher with PBE+D3 (full coverage, 5 H_2_O/Mg^2+^) than predicted by CCSD­(T) or experimentally observed.

This deficiency of PBE+D3 for isotherm predictions arises from the overestimation of H-bond strengths between the water molecules, as well as between the water molecules and the MOF framework, see Section 3.2. This is a known limitation of widely employed GGA-level functionals, such as PBE with D2 or D3 dispersion terms.^59,61–64^ In contrast, wave function-based electron correlation methods,^65,66^ such as MP2 ^65^ or CCSD­(T),^67,68^ yield results which agree within chemical accuracy limits with experiment.^69^ With our computational protocol, PBE+D3 yields a water dimerization energy of −24.9 kJ/mol and CCSD­(T) (at the PBE+D3 structure) yields −21.5 kJ/mol, see Section S2.3 in the SI. The Monte Carlo (MC) simulations of Siepmann and coworkers,^32^ which also use PBE+D3, predict much lower adsorbed amounts at a given pressure than our *Multisite Langmuir* isotherm model with PBE+D3, see Section S5.5 in the SI. Compared to experiments at 20 mbar, PBE+D3 Monte Carlo simulations underestimate the loading by about 1 H_2_O/Mg^2+^, whereas our PBE+D3 *Multisite Langmuir* model exceeds the experimental value by about 0.75 H_2_O/Mg^2+^.

For computational affordability, PBE+D3 or other functionals making use of the Generalized Gradient Approximation with some account of dispersion are commonly used when periodic structures with hundreds of atoms in the simulation cell are studied. Our results raise doubts that, if chemical accuracy is required, this level of DFT is suitable for Molecular Dynamics or Monte Carlo simulations for water in MOFs, or for generating machine learning potentials.^70^ The application of hybrid functionals seems within reach now, but will require careful and substantial testing if chemical accuracy is the target, see Section 2.4 of the SI.

The right panels of Figure 7 show two other experimental isotherms, one reported by Yaghi and coworkers^2^ and the other by Ann and coworkers.^29^ At 30 mbar, they exhibit a sorption capacity that is about 0.5 and 1.5 H_2_O/Mg^2+^, respectively, lower than the experimental isotherms^28,29^ shown in the left panel, and also lower than our simulations based on the *Multisite Langmuir* model for the ideal structure. However, agreement with our CCSD­(T) predictions can be achieved if we assume that only 87% and 69% of all sites are available for water adsorption in the samples used in refs 2 and 29, respectively. The strongest reduction of the sorption capacity (69%, blue triangles) is reported by Ahn and coworkers^29^ for a sample that was synthesized under sono-chemical conditions.^29^ One possible explanation for the large variations between the experimental isotherms is that the samples of refs 2 and 29 have structure imperfections such as blocked pores, missing linkers or partially protonated linkers. For CO_2_ adsorption on Ni- and Mg-MOF-74, experiments suggest that only 92% and 78%, respectively, of the metal ion sites are actually accessible, possibly because “the remaining metal sites are physically obstructed from access because of defects in the crystal structure”.^21^ Moreover, isotherm simulations for CH_4_ and CO_2_ adsorption in Mg-MOF-74 using the same methodology as applied here yielded close agreement with experiment assuming that only 78% and 76.5% of the Mg^2+^ sites, respectively, are accessible.^46,47^ Further, an NMR study has produced evidence for solvent-derived formate defects in Mg-MOF-74 and demonstrated that the CO_2_ uptake depends on the defect concentration.^71^ For the MOF UiO-66, low-dose high-resolution transmission electron microscopy (HRTEM) has shown that “missing-linker defects were observed in various [...] samples, including those that would have typically been assumed to be essentially defect-free, which underlines that notionally perfect materials can contain defects invisible to most characterization techniques that may influence observed variance in properties of MOFs such as gas uptake”.^36^

Another possible explanation for the reduced sorption capacity is that half (3) or all (6) of the 6 Mg^2+^ sites are blocked as the corresponding isotherm simulations, “0.5-A” and “0-A”, respectively, show. This blocking could be either due to incomplete activation of the synthesized material or due to incomplete water desorption before the adsorption measurements. This explanation is supported by the thermogravimetric (TG) experiment of Dietzel et al.^20^ for the fully water-loaded sample. After increasing the temperature to 423 K, 4 H_2_O/Mg^2+^ had left the sample, but a significantly higher temperature was needed, 623 K, to remove the fifth water molecule per Mg^2+^, see Figure 8.

**Figure 8:**
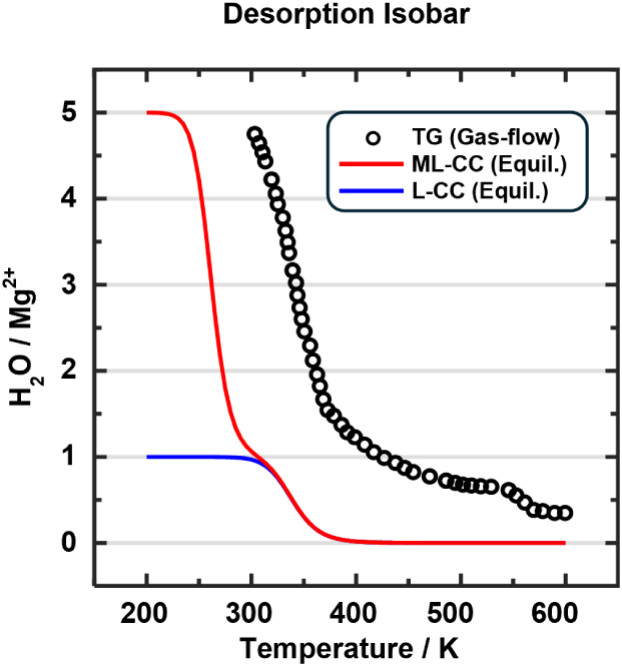
Predicted isobars assume equilibrium (5 Pa pressure) compared to results of a thermogravimetric (TG) experiment under air-flow conditions (black circles).^20^ Red line (ML-CC, Equil.)=*Two-step Langmuir* model starting at full loading; blue line (L-CC, Equil.)=*Langmuir model* for the last desorption step only, 1 H_2_O/Mg^2+^ → 0 H_2_O/Mg^2+^. CCSD­(T) corrected adsorption energies are employed.

We cannot directly model the flow conditions of the TG experiment, but we can calculate an isobar assuming equilibrium at a pressure of 5 Pa (*Two-step Langmuir* model, see Sections S5.2 and S5.6 in the SI). As Figure 8 shows, our predicted isobar reproduces the overall shape of the experimental TG curve well. At 305 K, four water molecules (located at sites **B** to **E**) have been removed, whereas removal of the last water molecules from the Mg^2+^ sites is completed only at about 400 K. This confirms that higher temperatures are needed to remove the most strongly bound water molecules located at **A** sites. In addition, the isobar corroborates the suggestion of Furukawa et al.^2^ that the activation condition used for the repeated adsorption measurements (298 K and 5 Pa) does not remove the last water molecule from the MOF pores.

## 5 Conclusions

DFT structure optimizations provide detailed atomistic understanding of how water molecules organize inside the pores of MOFs as a function of water loading.^8^ For adsorption of one to five water molecules per Mg^2+^ ion in the hexagonal pores of Mg-MOF-74, we have shown that in each step well-defined water structures, anchored to the Mg^2+^ ions on the internal MOF surface, form and retain the *R*3̅ symmetry of the MOF host. With 4 H_2_O/Mg^2+^, a two-dimensional monolayer is completed, covering the pore wall. For 5 H_2_O/Mg^2+^, the additional water molecules form a tube-like stack of water trimers inside the pores which are connected with H-bonds to the water monolayer at the pore walls. This creates a particularly stable structure, in which every water molecule is 4-fold coordinated and stabilized by 3.8 H-bonds on average.

Whereas DFT (PBE+D3) yields reliable structures, adsorption energies obtained at this level are not accurate enough for isotherm predictions. As already shown in previous studies for other small molecules,^25,46,72^ employing Coupled Cluster theory, CCSD­(T), within a *hybrid QM:QM approach* ^24^ yields isotherms for water adsorption in the ideal Mg-MOF-74 host that are in close agreement with measured isotherms for supposedly defect-free samples.^28,29^ We have reached a level of agreement that corresponds to ±2 kJ/mol deviation of the calculated Gibbs free energies from experiment. With our *Multisite Langmuir* model we are able to connect the contribution of each loading state to the total isotherm of the system.

We further show that agreement with reported isotherms^2,29^ which do not reach full loading with increasing pressure is achieved if we assume (i) that only about 70 to 90% of the adsorption sites of the ideal structure are accessible, or (ii) that water had not been completely removed from the Mg^2+^ sites before measurement. This suggests that the observed significant variations among experimental isotherms likely stem from sample imperfections and/or incomplete evacuation. Our results confirm that quantum chemical isotherm predictions have reached a level of accuracy that deviations between predictions and experiments indicate variations in the samples or sample imperfections.^25,46,72^

## Supplementary Material





## Notes

A separate file provides the optimized structures and example inputs (ZIP)
Details of computational methods and models; lattice vectors; optimized adsorption structures; adsorption energies and thermodynamic data; an analysis of alternative pore-filling schemes; results of adsorption isotherm/isobar simulations: multistep Langmuir model; comparisons to experimental and computational results (PDF)

## §

University of Cambridge, Yusuf Hamied Department of Chemistry, Lensfield Rd, Cambridge CB2 1EW, United Kingdom

## ∥

Freudenberg Technology Innovation SE & Co. KG, Hoehnerweg 2–4, 69469 Weinheim, Germany
