# Supplementary material for: Atomistic Model for Water Adsorption in Mg-MOF-74: Quantum Chemical Prediction of Structures and Isotherms
Source: J Am Chem Soc. 2026 Mar 16;148(11):12296–306. doi: 10.1021/jacs.6c01686 (PMC13022884; doi:10.1021/jacs.6c01686)
Supplement: Supplementary file 2 [file ja6c01686_si_002.pdf]

Supporting Information

Atomistic Model for Water Adsorption in Mg-MOF-74: Quantum Chemical Prediction of Structures and Isotherms

Nicole Mancini,[a] Fabian Berger,[a†*] Marcin Rybicki,[a‡] Kaido Sillar,[b*] and Joachim Sauer[a*]

[a] Institut für Chemie, Humboldt-Universität zu Berlin, Unter den Linden 6, 10117 Berlin, Germany

[b] University of Tartu, Institute of Chemistry, Ravila 14a, 50411 Tartu, Estonia

* Corresponding authors email adresses:
Fabian Berger, email: fabian.berger@chemie.hu-berlin.de
Kaido Sillar, email: kaido.sillar@ut.ee
Joachim Sauer, email: js@chemie.hu-berlin.de

† Present address: University of Cambridge, Yusuf Hamied Department of Chemistry, Lensfield Rd, Cambridge CB2 1EW, United Kingdom

‡ Present address: Freudenberg Technology Innovation SE & Co. KG, Hoehnerweg 2-4, 69469 Weinheim, Germany

Table of Contents

S1 LATTICE OPTIMIZATION 3

S2 MODELS AND METHODS 7

S2.1 PBE+D3 Calculations with periodic boundary conditions 7

S2.2 CCSD(T) Calculations on Cluster Models 8

S2.3 Structure and Binding Energy of the Water Dimer 11

S2.4 DFT-D Calculations with Hybrid Functionals for Water in Mg-MOF-74 12

S3 ADSORPTION STRUCTURES 14

S3.1 Symmetric Structures 14

S3.1.1 Loading 1 H_2O/Mg^{2+} – Site A 15

S3.1.2 Loading 2 H_2O/Mg^{2+} – Site AB 16

S3.1.3 Loading 2 H_2O/Mg^{2+} – Site AD 18

S3.1.4 Loading 3 H_2O/Mg^{2+} – Site ABC 20

S3.1.5 Loading 4 H_2O/Mg^{2+} – Site ABCD 22

S3.1.6 Loading 5 H_2O/Mg^{2+} – Site ABCDE 24

S3.2 Additional Structures 27

S3.2.1 Loading 0–1 H_2O/Mg^{2+} 27

S3.2.2 Summary of additional Structures 28

S4 ADSORPTION ENERGIES AND THERMODYNAMICS 30

S4.1 Thermodynamic Quantities per Water Molecule 30

S4.2 Results at 313 K 32

S4.3 Loading 0–1 H_2O/Mg^{2+} 34

S4.4 Comparison Between Space Group P1 and $R\overline{3}$ PBE+D3 Results 35

S5 ADSORPTION ISOTHERMS AND ISOBARS 36

S5.1 Deviation Between *Multi-site Langmuir* Predictions and Experiments....36

S5.2 *Multi-step* Langmuir Model 37

S5.3 Scaling and Preoccupation Effects on Isotherm Simulations 39

S5.4 Scaling Experimental Isotherms 41

S5.5 Comparison with Monte Carlo Simulation and DFT-Derived Force-Field 42

S5.6 *Multi-site*, *Multi-step* and *Two-step* Langmuir Desorption Isobars 44

S6 REFERENCES 45

S1. LATTICE OPTIMIZATION

The conventional unit cell and the rhombohedral Niggli-reduced cell are shown in Figure S1.1, and the lattice parameters are reported in Table S1.1.

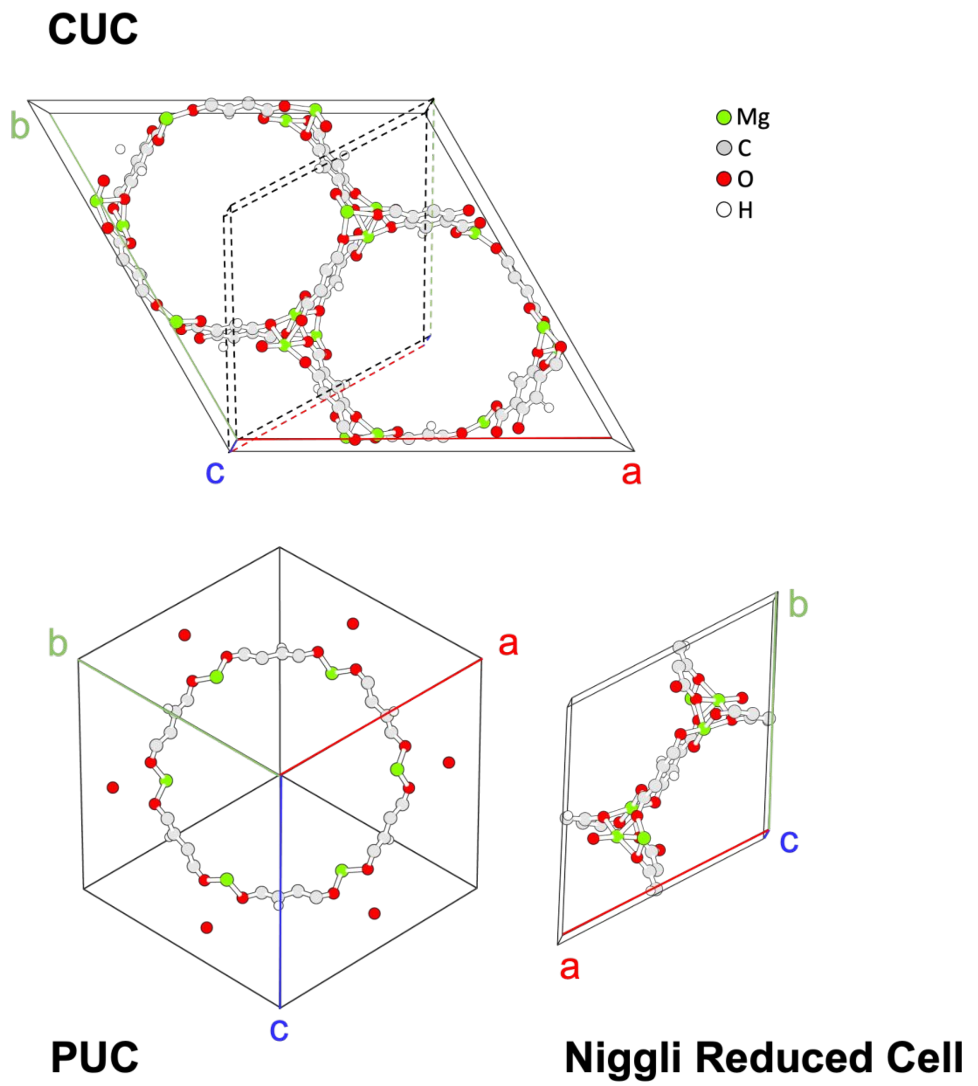

Figure S1.1. Conventional unit cell (top), primitive unit cell – PUC (bottom left), and Niggli reduced cell (bottom right and inside the conventional) of Mg-MOF-74. Color code: magnesium – light green; carbon – gray; oxygen – red; and hydrogen – white.

Table S1.1. Cell vector lengths, a, b, and c [Å], and angles, α, β, and γ [°] as well as the number of Mg^{2+} ions and $(dobdc)^{4-}$ linkers for the the primitive unit cell (PUC), the Niggli reduced cell (NRC), and the conventional unit cell (CUC), based on the CUC structure optimized at PBE+D3 level.

	PUC	NRC	CUC[1]
Mg^{2+}	6	6	18
$(dobdc)^{4-}$	3	3	9
a	15.235	15.235	26.084
b	15.235	15.235	26.084
c	15.235	6.917	6.917
α	117.8	117.8	120.0
β	117.8	98.7	90.0
γ	117.8	98.7	90.0

The DFT-D cell parameters obtained for the bare CUC of Mg-MOF-74 are in good agreement with the experimentally measured lattice constants reported in Table S1.2.

Table S1.2. Cell vector lengths a, b and c [Å] for the conventional unit cell of Mg-MOF-74 obtained with powder x-ray diffraction (PXRD), neutron powder diffraction (NPD), and optimized with PBE+D3 obtained at temperature *T* [K].

Method[a]	Reference	*T*	a = b	c
PBE+D3	This work	0	26.084	6.917
NPD	Yaghi 2011[1]	20	25.921(2)	6.8625(8)
PXRD	Yildirim 2008[2]	20	25.892(2)	6.874(1)
NPD	Yildirim 2010[3]	20	25.88(12)	6.8789(5)
PXRD	Matzger 2008[4]	298	26.02(1)	6.721(4)
NPD	Long 2014, [1,5] Yaghi 2011[33]	298	25.9111(20)	6.8687(12)

[a] All experiments assume the $R\overline{3}$ space group.

The PBE+D3 a = b, and c values are 0.2% to 0.8%, and 0.6% to 2.9%, respectively, larger than the experimental values. Yaghi and co-workers observed a significant expansion along the a/b-axes and a simultaneous compression in the c-axis for the bare Mg-MOF-74 upon increasing temperature.[33] When we compare our results with their low-temperature (20 K) measurements, our values a = b and c are 0.6% and 0.8%, respectively, larger than the experimental data. In our PBE+D3 optimizations an increase of the cell volume by 0.34% is observed upon adsorption of one H_2O molecule on all Mg^{2+} sites, that is, 1 H_2O/Mg^{2+} site (18 H_2O/CUC = 6 H_2O/PUC) and a contraction in volume of 0.5% upon the adsorption of 5 H_2O/Mg^{2+}.

Table S1.3. a, b and c CUC-cell parameters [Å] for $Mg_2(dobdc)\cdot(H_2O)_{10}$ structure obtained with powder x-ray diffraction (PXRD) and optimized with PBE+D3 obtained at the temperature *T* [K].

Method	Reference	*T*	a = b	c
PXRD	Fjellvåg 2008[6]	298	26.02607(6)	6.758722(30)
PBE+D3	This work	0	25.918	6.70

Table S1.4. a,b,c Niggli-reduced cell length parameters [Å] and α, β, and γ angles [°] parameters for loading 0 to 5 H_2O/Mg^{2+} optimized at PBE+D3 in space group *P1*.

H_2O/Mg^{2+}	α	β	γ	a	B	c
0	117.8	98.7	98.7	15.238	15.238	6.917
1	117.6	98.9	98.8	15.131	15.191	7.019
2	118.3	99.1	98.5	15.126	15.264	6.958
3	117.9	98.5	89.4	15.098	15.107	6.670
4	117.9	98.5	98.5	15.225	15.225	6.752
5	117.9	98.4	98.4	15.279	15.278	6.710

Table S1.5. a,b,c Niggli-reduced cell length parameters [Å] and α, β, and γ angles [°] parameters for loading 0 to 5 H_2O/Mg^{2+} optimized at PBE+D3 level in space group $R\overline{3}$.

H_2O/Mg^{2+}	α	β=γ	a=b	c
0	117.8	98.7	15.236	6.918
1	117.5	98.9	15.170	7.016
2	118.0	98.2	15.327	6.595
3	117.9	98.5	15.121	6.690
4	117.9	98.5	15.149	6.694
5	117.9	98.4	15.046	6.612

S2. MODELS AND METHODS

We have developed an ab initio approach for predicting adsorption isotherms based on a "local" adsorption model that combines DFT-D with highly accurate *ab initio* calculation.[7,8]

S2.1 PBE+D3 Calculations with periodic boundary conditions

For both atom position optimizations and Hessian calculations, a 500 eV energy cutoff is used. The calculations are performed at the Γ-point only. Further information about the simulation cells as well as lattice vector and atom position optimizations are given in Section S1. To minimize the effect of volume changes on the basis sets, cell shape and size optimizations are performed using an 800 eV kinetic energy cutoff. PBE+D3 Hessians of the entire system (including framework vibrations) are calculated numerically using central finite differences with Cartesian displacements of 2 pm and an energy convergence threshold of 10^{-8} eV. The MonaLisa code[9,10] is used to calculate zero-point vibrational energies, as well as thermal enthalpy and entropy contributions from harmonic wavenumbers. For molecular dynamics (MD) simulations, an energy cutoff of 400 eV and an energy convergence threshold of 10^{-5} eV are employed, using the 'normal' precision setting instead of the 'accurate' setting used otherwise. Simulations are performed in the NVT ensemble using a Nosé–Hoover thermostat[11,12] with a time step of 1.0 fs. For simulated annealing, the temperature is increased from 400 to 1000 K with a ramp of 0.025 K/fs and subsequently cooled to 200 K using the same rate. The final structure is then relaxed using the consistent optimization settings described above.

The model of the Mg-MOF-74 is obtained from the Cambridge Crystal Database (CSD). Starting from the experimental structure, which does not contain hydrogen atoms, hydrogen atoms are added to the benzene ring of the $(dobdc)^{4-}$ linker and to the oxygen atoms of the water molecules.

For the two experimentally available crystal structures, $Mg_2(dobdc)$ and $Mg_2(dobdc)\cdot(H_2O)_{10}$, optimizations at PBE+D3 level were performed using the conventional unit cell (CUC) in the space group $R\overline{3}$. Two types of optimizations were performed consecutively: a full optimization of both atomic positions and lattice vectors were conducted iteratively until converge was reached, followed by an optimization of the atom positions only, with the lattice constants fixed to the optimized values. The DFT-optimized structures (atoms and lattice constants) are compared to experimental atom positions and lattice constants.

All further PBE+D3 calculations are carried out in a Niggli-reduced rhombohedral unit cell containing 6 Mg^{2+} ions connected with 3 $(dobdc)^{4-}$ organic linkers, resulting in a total of 54 atoms per simulation cell for the bare MOF and up to 144 atoms in the case of the fully loaded system. This cell is derived from the CUC of the $Mg_2(dobdc)\cdot(H_2O)_{10,}$ containing 18 Mg^{2+} ions,[13] which is converted into a primitive unit cell (PUC) containing 6 Mg^{2+} and further transformed via the Niggli algorithm.[14,15] All optimizations are conducted with reinforcement of the symmetry inherent to the crystal. Figure S1.1 and Table S1.1 show and compare the composition of the Niggli reduced cell employed with CUC and PUC. For the optimization of a gas-phase water molecule, a large cubic cell (a = b = c = 15 Å; α = β = γ = 90.0°) with the same cut-off energy and all the other parameters is used.

S2.2 CCSD(T) Calculations on Cluster Models

We calculate all the pairwise H-bond interactions between water molecules and between water molecules and Mg and linker sites of Mg_2(dobdc) with CCSD(T). We also include interactions with O–O distances slightly beyond 350 pm for which we find a significant high-level correction. Figure S2.1 shows the cluster models employed, with H_2O and linker molecules in ball-and-stick representation. Dangling bonds on O and C atoms are saturated by adding hydrogen atoms at constant bond distances of 95.3 pm (O–H) and 109.0 pm (C–H), respectively.[16]

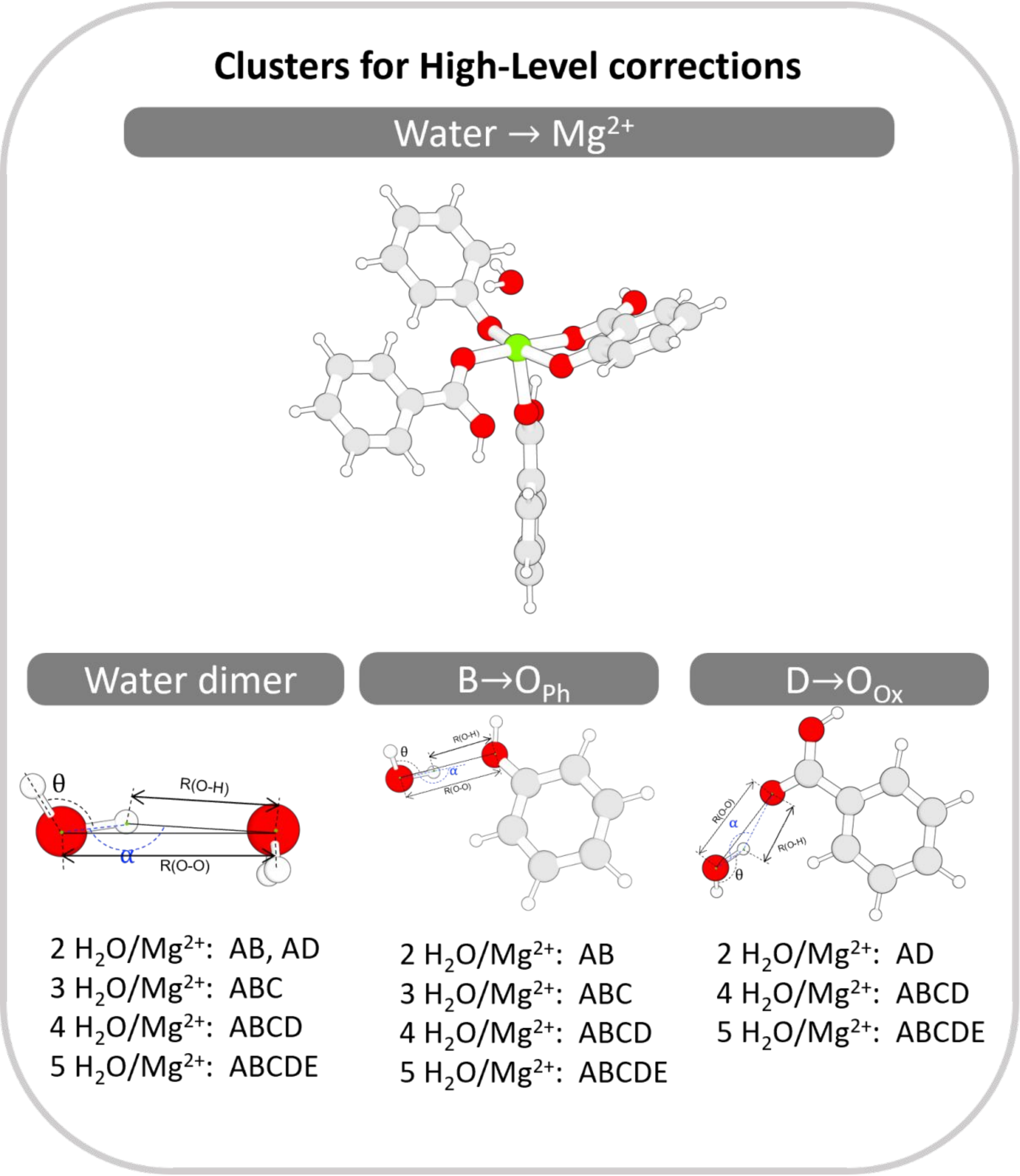

Figure S2.1. Cluster scheme for high-level correction (ΔCC) of H-bonds in various motifs. **Top**: Interactions between water and Mg^{2+}. **Bottom**: Interactions include water–water (left, loading 2 to 5 H_2O/Mg^{2+}), B–O_{Ph} – phenolic-linker (center, loading 2 H_2O/Mg^{2+} sites AB and loading 3 to 5 H_2O/Mg^{2+}), and D–O_{Ox} – carboxylic-linker (right, loading 2 H_2O/Mg^{2+} sites AD and loading 4 H_2O/Mg^{2+}). Color code: magnesium – light green; carbon – gray; oxygen – red; and hydrogen – white.

The high-level cluster calculations are performed using the domain-based local pair natural orbital (DLPNO) CCSD(T) method[17] as implemented in the ORCA program[18,19] version 4.2.1. All cluster calculations are counterpoise (CP)[20] corrected for basis set superposition errors (BSSE), and the energies are extrapolated to the complete basis set limit using a two-point extrapolation scheme[21,22] for aug-cc-pV*X*Z basis sets with *X* = T, Q.[23,24] The settings "TightPNO" and "TightSCF" are used for DLPNO calculations. Low-level, PBE+D3 cluster calculations employ def2-TZVP basis sets,[25] and the energies are also CP[20] corrected. The interaction between a water molecule and a Mg^{2+} cation is calculated with the same method.

The inclusion of diffuse functions in the basis set has an impact on adsorption energies. However, a full basis set extrapolation using augmented basis sets (aug-cc-pV*X*Z) with *X* = T, Q is computationally prohibitive for the water-framework interaction, where a large cluster representing the MOF is needed for an accurate description. For the water–MOF cluster, we employ a basis set extrapolation from aug-cc-pVDZ to aug-cc-pVTZ instead. The correction due to diffuse functions is approximated by computing the adsorption energy difference between cc-pV*X*Z and aug-cc-pV*X*Z basis sets with *X* = D,T, defining a correction term ΔAUG,

$$\Delta\mathrm{AUG} = \Delta\mathrm{CC}^{\mathrm{AUG(D,T)}} - \Delta\mathrm{CC}^{\mathrm{STD(D,T)}}. \qquad \text{(S.2.1)}$$

This correction is added to the *X* = T, Q extrapolated adsorption energy as obtained from the basis sets without augmented diffuse basis functions (cc-pV*X*Z), $\Delta CC^{STD(T,Q)}$, yielding the final correction:

$$\Delta\mathrm{CC}^{\mathrm{AUG(T,Q)}} = \Delta\mathrm{CC}^{\mathrm{STD(T,Q)}} + \Delta\mathrm{AUG} \qquad \text{(S.2.2)}$$

To assess the reliability of pairwise high-level correction (ΔCC) evaluated at PBE+D3BJ structures we calculate the dissociation energy of a water tetramer using a many-body decomposition and compare our results with literature data.[26]

The total dissociation energy obtained with our settings agrees with the benchmark values within 0.1 kJ/mol per water molecule, and the 2-, 3-, and 4-body terms agree within 0.3, 0.2, and 0.0 kJ/mol per water molecule, respectively. The many-body decomposition shows that the three-body contribution is indeed important for the dissociation energy, accounting for about 20% of the total. In contrast, the high-level (HL) correction is already accurately captured by pairwise interactions (8.1 of a total 8.2 kJ/mol per water), and higher-order terms contribute less than 0.3 kJ/mol. This demonstrates that while three-body terms are essential for the total dissociation energy, their effect is well described by DFT, and it is therefore sufficient to correct only the two-body interactions at the CCSD(T) level.

These findings validate our approach, which accounts for higher-order contributions through DFT calculations with periodic boundary conditions (pbc), using the PBE+D3 functional, and adds CCSD(T) corrections based on pairwise interactions. The individual contributions to the dissociation energy are shown in Table S2.1.

Table S2.1. Dissociation energies (E_{diss}) and their many-body contributions (two-body terms $E_{2\text{-body}}$, three-body terms $E_{3\text{-body}}$, and four-body terms $E_{4\text{-body}}$) per water molecule, in kJ/mol. Interaction energies are calculated with respect to unrelaxed individual water molecules. Values are shown for structures optimized using RI-MP2/aug-cc-pVDZ,[26] B3LYP+D3/def2-TZVP, and PBE+D3BJ/def2-TZVP, where BJ stands for Becke–Johnson damping. The high-level (HL) correction corresponds to the difference between CCSD(T) and the respective DFT-D functional for the interaction energies.

	E_{diss}	$E_{2\text{-body}}$	$E_{3\text{-body}}$	$E_{4\text{-body}}$
**MP2 structure[a]**				
CCSD(T)[a]	30.2[a]	23.1[a]	6.5[a]	0.6[a]
CCSD(T)	30.3	23.4	6.3	0.6
PBE+D3BJ	38.5	31.6	6.0	0.9
HL(CCSD(T)–PBE+D3BJ)	8.2	8.1	-0.2	0.3
**PBE+D3BJ structure**				
CCSD(T)	32.2	22.5	8.7	0.9
B3LYP+D3	39.0	28.7	9.3	1.0
HL(CCSD(T)–B3LYP+D3)	6.8	6.1	0.5	0.2
PBE+D3BJ	41.6	31.6	8.7	1.3
HL(CCSD(T)–PBE+D3BJ)	9.5	9.1	0.0	0.4
**B3LYP+D3 structure**				
CCSD(T)	30.6	23.2	6.7	0.6
B3LYP+D3	37.1	29.3	7.0	0.8
HL(CCSD(T)–B3LYP+D3)	6.5	6.1	0.3	0.1

[a] Ref.[26]

Dissociation energies calculated with B3LYP+D3 are similar to the PBE+D3BJ results obtained using the PBE+D3BJ structures, varying by less than 3 kJ/mol per water molecule. While the B3LYP+D3 dissociation energies come closer to the CCSD(T) results, there remains a significant discrepancy of 6.8 kJ/mol per water molecule. This suggests that B3LYP+D3 does not provide a significant improvement over PBE+D3BJ in terms of dissociation energies, which agrees with previous literature,[27] and, thus, does not justify the increased computational demand of B3LYP+D3 with pbc over PBE+D3BJ with pbc.

A comparison of dissociation energies based on structures optimized with RI-MP2/aug-cc-pVDZ,[26] B3LYP+D3/def2-TZVP, and PBE+D3BJ/def2-TZVP shows that the corresponding CCSD(T) dissociation energies differ by no more than 0.4 or 2 kJ/mol per water molecule when optimized with B3LYP+D3 or PBE+D3BJ, respectively. This suggests that B3LYP+D3 structures are more similar to the MP2 structures than the PBE+D3 structures, also in agreement with the literature,[27] but the error introduced in the CCSD(T) dissociation energy is sufficiently small and remains within the uncertainty limits proposed in this work. The relatively small structural changes constitute another argument, supporting the use of PBE-D over B3LYP-D in our calculations with pbc.

S2.3 Structure and Binding Energy of the Water Dimer

To further benchmark our methodology against experimental[28] and computational literature (MP2[29]and CCSD(T)[30]), we optimize a water dimer and calculate the dimerization energy. The initial structure is taken from the ice I_h crystal structure[31] and optimized in the gas phase with the PBE+D3 functional, following the same procedure as for the water monomer, in a cubic 15 Å cell with identical computational parameters (see Section S2.1). A single point CCSD(T) calculation is performed on the optimized structure to obtain the high-level correction to the electronic dimer formation energy, as described in Section S2.2. The O···O distance, hydrogen-bond angle, and dimer formation energy are summarized in Table S2.2.

Our CCSD(T):PBE+D3 interaction energy (−21.5 kJ/mol) agrees well with previous CCSD(T) results (−20.9 kJ/mol) and falls within the experimental range (−22.6 ± 2.9 kJ/mol), supporting the reliability of our method to describe H-bonding in the water-dimer.

Table S2.2. Intermolecular O···O distance *R* (pm), hydrogen-bond angle α (°), and interaction energy Δ*E* (kJ/mol) of the water dimer from this work, compared with selected experimental and computational literature values. The structure is optimized with PBE+D3 and energies are computed at the PBE+D3 and CCSD(T) levels.

Ref.	Method	*R*(O···O)	α	Δ*E*
This work	PBE+D3	289	171.3	–24.9
	PBE+D3 + ΔCC[a]	289[a]	171.3[a]	–21.5[a]
Helgaker[30]	CCSD(T)	289	172.9	–20.9
Dixon[29]	MP2	–	–	–20.5
Blander [28]	Experiment	295	171.1	–22.6±2.9

[a] Single point at PBE+D3-optimized structure

S2.4 DFT-D Calculations with Hybrid Functionals for Water in Mg-MOF-74

For loadings of n = 1 to 5 H_2O/Mg^{2+} in Mg–MOF-74, $Mg_2(dobdc)\cdot(H_2O)_{2n}$, we calculated single point adsorption energies at the PBE+D3 structures with the HSE06+D3,[32] PBE0+D3,[33-35] and B3LYP+D3[36-38] hybrid functionals as implemented in the Vienna Ab Initio Simulation Package (VASP) version 6.2.0.[39,40] We used the original implementation of D3, which is also often referred to as 'zero-damping'. We followed the same protocol as for the PBE+D3 calculations described in Section S2.1. Moreover, we reoptimized the adsorption structures with B3LYP+D3.

Table S2.3 shows single point adsorption energies per water molecule for loadings of n H_2O/Mg^{2+} obtained with different hybrid functionals at the PBE+D3-optimized structures. The PBE+D3 + ΔCC results serve as reference. They are accurate within ±2 kJ/mol as the agreement with the experimental adsorption isotherms indicates. ΔCC are the CCSD(T) high-level corrections. Table S2.4 compares the PBE+D3 and B3LYP+D3 adsorption energies, Δ*E*, for the optimized structures.

Table S2.3. Single point adsorption energies, Δ*E*, per water molecule for loadings of n H_2O/Mg^{2+}, obtained with different hybrid functionals at the PBE+D3-optimized structures and their differences to the PBE+D3 + ΔCC reference energies, Δref, all in kJ/mol.

n	PBE+D3 + ΔCC	PBE0+D3		HSE06+D3		B3LYP+D3	
	Δ*E*	Δ*E*	Δref	Δ*E*	Δref	Δ*E*	Δref
1	−79.8	−85.4	−5.6	−81.4	−1.6	−85.0	−5.2
2	−76.8	−82.3	−5.5	−78.9	−2.1	−84.2	−7.4
3	−72.8	−79.2	−6.4	−76.6	−3.8	−80.5	−7.7
4	−72.5	−78.3	−5.8	−76.4	−3.9	−79.6	−7.1
5	−74.2	−81.1	−6.9	−79.7	−5.5	−81.9	−7.7

Table S2.4. Adsorption energies per water molecule for loadings of n H_2O/Mg^{2+}, Δ*E*, obtained with PBE+D3 and B3LYP+D3 structure optimization. Also given are the differences between the B3LYP+D3 and PBE+D3 energies, ΔΔ*E*, and the difference with respect to the PBE+D3 + ΔCC reference energies, Δref, all in kJ/mol.

	PBE+D3		B3LYP+D3		
n	Δ*E*	Δ*E* + ΔCC	Δ*E*	ΔΔ*E*	Δref
1	−80.1	−79.8	−84.1	−4.0	−4.3
2	−78.3	−76.8	−83.3	−5.0	−6.5
3	−76.6	−72.8	−79.9	−3.3	−7.1
4	−76.7	−72.5	−79.2	−2.5	−6.7
5	−79.6	−74.2	−81.2	−1.6	−7.0

For PBE0+D3 and B3LYP+D3 single point calculations (Table S2.3), the deviations of adsorption energies from the references increase with increasing loading from −5.6 to −6.9 kJ/mol and from −5.2 to −7.7 kJ/mol, respectively. With the range separated HSE06+D3 functional, the deviations are smaller, increasing from −1.6 to −5.5 kJ/mol. The deviations also become slightly smaller for B3LYP+D3 optimized structures, from −4.3 to −7.0 kJ/mol with increasing loading, see Table S2.4. All these deviations are outside the chemical accuracy range of ±4 kJ/mol.

Table S2.4 shows that B3LYP+D3 with pbc overestimates the binding even more than PBE+D3. The difference of the electronic energies, $\Delta\Delta E$, is −4.0 kJ/mol for the H_2O-Mg^{2+} interaction (n = 1) and changes from −5.0 to −1.6 kJ/mol with increasing loading from n = 2 to 5. It is known that B3LYP+D3 does not improve adsorption energies for water compared to PBE-D.[27] This analysis provides further justification for our approach to use PBE+D3-based structures and low-level energies, and to correct the adsorption energies using CCSD(T)-quality pairwise interaction energies.

S3. ADSORPTION STRUCTURES

S3.1 Symmetric Structures

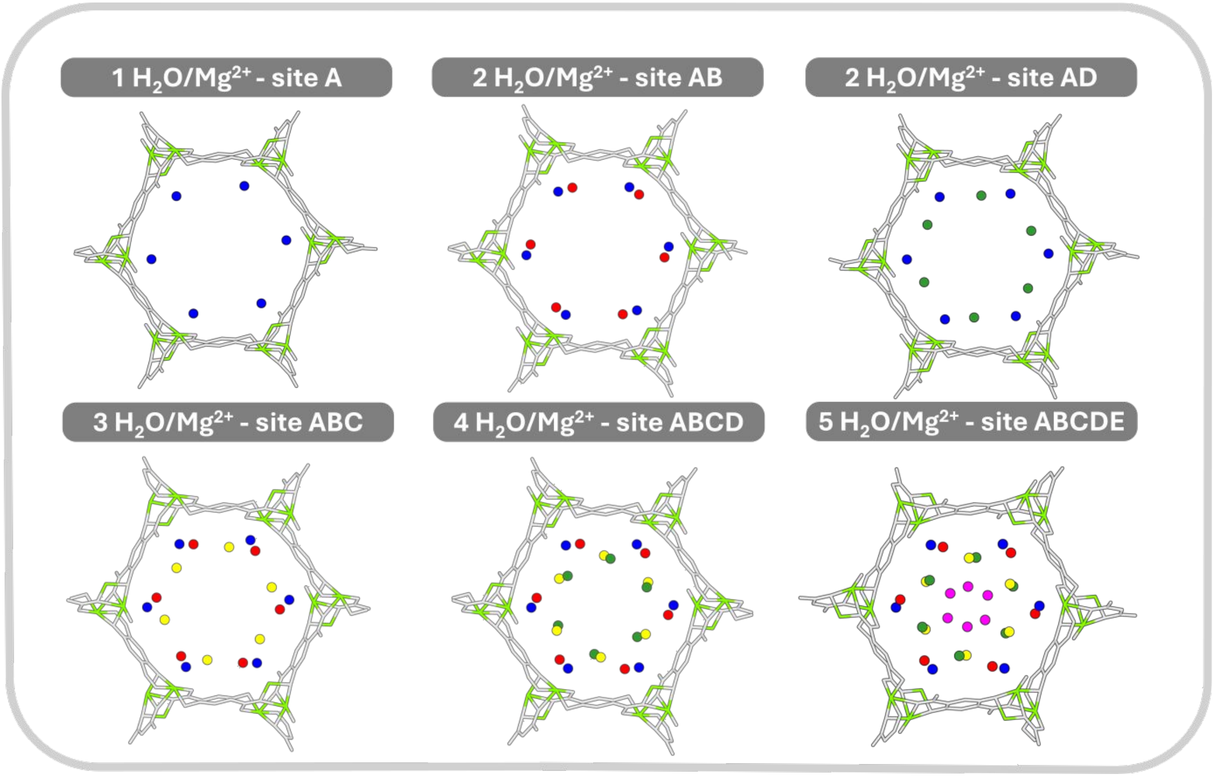

Figure S3.1. View on six pores of DFT-optimized $Mg_2(dobdc)\cdot(H_2O)_{2n}$ with n = 1, 2, 3, 4, and 5. Color code: carbon and oxygen of $(dobdc)^{4-}$ linker – gray; magnesium – light green; water adsorbed at site A (Mg^{2+}) – blue; at site B–red; at site C – C-yellow; at site D – forest green; and at site E – magenta.

Initial structures for loadings of 5 to 0 H_2O/Mg^{2+} are obtained by removing one type of symmetry-equivalent oxygen atom from the experimental structure[6] in each step and by adding hydrogen atoms to both the oxygen atoms of the water molecules and the benzene carbon atoms of the linker. Hydrogen atoms are placed next to the carbon atoms of the linker site at a distance of 109.0 pm within the plane of the benzene ring.

S3.1.1 Loading 1 H_2O/Mg^{2+} – Site A

The DFT-optimized structure with 1 H_2O/Mg^{2+} shows the magnesium–oxygen distance (Mg–O) of 218 pm and the Mg–O–H angle of 109.0°. The H–O–H angle of water molecules at site A is 104.7°, and the O–H distance is 100.00 pm. The six consecutive Mg^{2+} adsorption sites form a six-membered ring in a chair-conformation with an Mg–Mg distance of 807 pm. Similarly, the oxygen atoms of water molecules at site A are also oriented in a chair-shaped configuration with an O–O distance of 573 pm (Figure S3.2).

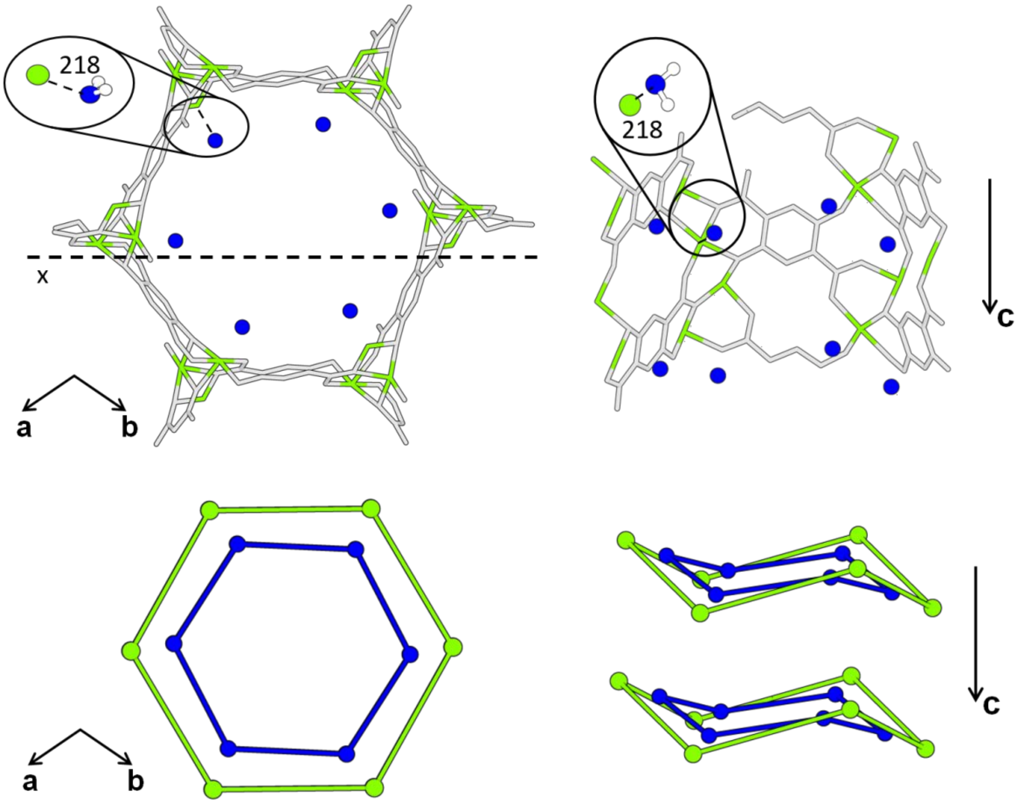

Figure S3.2. DFT-optimized structure (space group $R\overline{3}$) of $Mg_2(dobdc){\cdot}(H_2O)_2$. **Top left:** View in pore direction. **Top right:** View on the pore wall obtained by cutting the pore in halves by x axes and rotating 90° around the same axes. **Bottom**: Two schematic views on the hexagons formed by the Mg^{2+} ions and the oxygen atoms of the water molecules. Distances in pm. Color code: carbon and oxygen of $(dobdc)^{4-}$ linker – gray; magnesium – light green; and water adsorbed at site A (Mg^{2+}) – blue. Symmetry equivalent sites are connected by lines for visual guidance. The lines do not indicate the H-bond network.

S3.1.2 Loading 2 H_2O/Mg^{2+} – Site AB

The first configuration for loading 2 H_2O/Mg^{2+} consists of a water dimer adsorbed at the Mg^{2+} site, forming a strong H-bond between sites A and B. Another H-bond is formed between the water at site B and the phenolic oxygen of the $(dobdc)^{4-}$ linker. The H-bond details for this optimized structure are reported in Table S3.1. This structural motif is obtained by removing water molecules at sites C, D, and E (indicated in yellow, green, and magenta, respectively) from the experimental structure[6] (main text, Figure 4 – bottom right), adding hydrogen atoms to $(dobdc)^{4-}$ linker and O atoms of the waters (sites A and B), and subsequently optimized at PBE+D3 level. Unlike loading 1 H_2O/Mg^{2+}, the oxygen atoms of water molecules at sites A adopt a planar hexagonal configuration with a distance of 574 pm. The second water molecules are arranged in a chair-like hexagonal ring above the plane defined by the water molecules at site A with a distance of 565 pm inside the MOF pore, as illustrated in Figure S3.3.

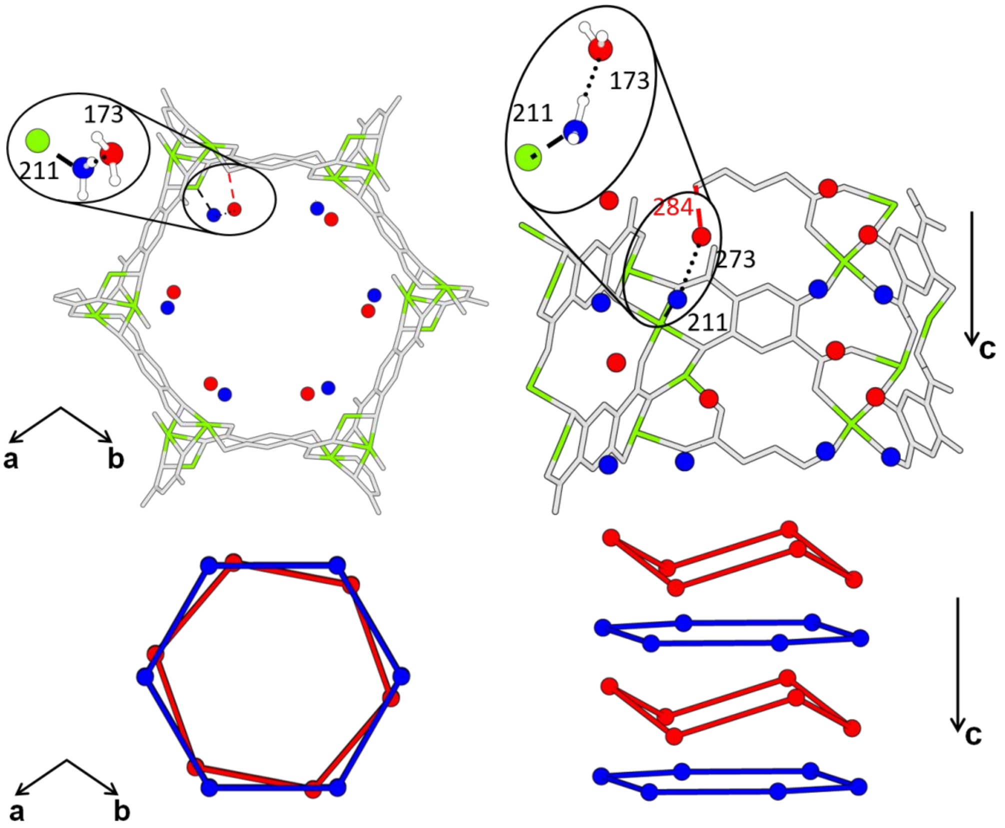

Figure S3.3. Top: Two views of DFT-optimized $Mg_2(dobdc)\cdot(H_2O)_4$ and a cluster of water showing details of interaction between water molecule at site A, Mg^{2+}, at site B and with the $(dobdc)^{4-}$ linker (dashed red line). **Bottom**: Arrangement of water molecules at site A and B. Distances in pm. Color code: carbon and oxygen of $(dobdc)^{4-}$ linker – gray; magnesium – light green; water adsorbed at site A (Mg^{2+}) – blue; and at site B – red. Symmetry equivalent sites are connected by lines for visual guidance. The lines do not indicate the H-bond network.

Table S3.1. Details of H-bonds for loading *n* = 2 H_2O/Mg^{2+} (sites AB): number, donor and acceptor sites, distances *R* [pm] and O–H–O (α) angle [°] as well as pair interaction energy, Δ*E* [kJ/mol] at PBE+D3 and CCSD(T) level.

Site	Color	Number (don, acc)	H-bond	*R*(O···O)	*R*(H···O)	α	Δ*E*	
							PBE+D3	CCSD(T)
A (Mg^{2+})	Blue	1 (1, 0)	A→B	273	173	174.8	–17.9	–15.1
B	red	2 (1, 1)	B→O_{Ph}	284	184	171.1	–15.2	–15.1

S3.1.3 Loading 2 H_2O/Mg^{2+} – Site AD

A second motif is tested in the case of loading 2 H_2O/Mg^{2+} for Mg-MOF-74. This exploration is done to check which configuration is more stable: water molecules located at sites A and B, or A and D. Indeed, both sites, B or D, can be independently filled after the occupation of A sites (Mg^{2+} sites). The second configuration for loading $2H_2O/Mg^{2+}$ consists of a sequence of hydrogen-bonded A and D sites running perpendicular to the pore direction (–A–D–A–D–) (Figure S3.10). A strong H-bond is formed between water molecules at sites A (donor) and D (acceptor). Additionally, a weaker H-bond is formed between the water at site D (donor) and the consecutive water at site A (acceptor). Finally, another H-bond is formed between water at site D (donor) and the carboxylic oxygen atom of the linker. As for loading 2 H_2O/Mg^{2+} site AB, water molecules at site A lay on a planar 6-membered ring with a distance of 571 pm while the second water molecules at site D are arranged in a chair-like hexagonal ring, with a distance of 551 pm, inside the sites A ring as shown in Figure S3.10. H-bond details for loading 2 H_2O/Mg^{2+} site for AD are reported in Table S3.5. However, as this structure is less stable than site AB, it is not considered in the isotherm calculations.

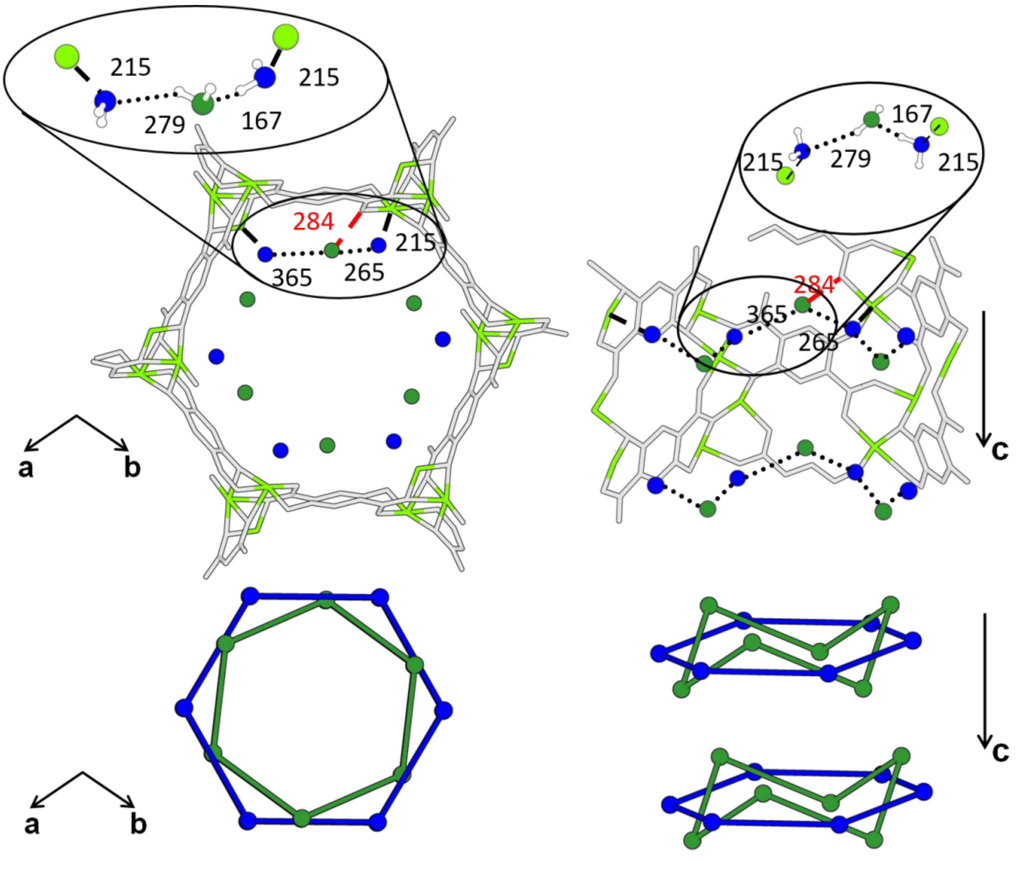

Figure S3.10. Top: Arrangement of water molecules from the DFT-optimized $Mg_2(dobdc)\cdot(H_2O)_4$ and a cluster of water showing details of interaction between water molecule at site A, Mg^{2+}, at site E and with the $(dobdc)^{4-}$ linker (dashed red line). **Bottom**: Arrangement of water molecules at site A and E. Distances in pm. Color code: carbon and oxygen of $(dobdc)^{4-}$ linker – gray; magnesium – light green; water adsorbed at site A (Mg^{2+}) – blue; and at site D – forest green. Symmetry equivalent sites are connected by lines for visual guidance. The lines do not indicate the H-bond network.

Table S3.5. Details of H-bonds for loading *n* = 2 H_2O/Mg^{2+} (site AD): number, donor and acceptor sites, distances *R* [pm] and O–H–O (α) angle [°] as well as pair interaction energy, Δ*E* [kJ/mol] at PBE+D3 and CCSD(T) level.

site	Color	Number (don, acc)	H-bond	*R*(O···O)	*R*(H···O)	α	Δ*E*	
							PBE+D3	CCSD(T)
A (Mg)	Blue	1 (1, 0)	A→D	265	167	11	–16.8	–13.3
II	Green	3 (2, 1)	D→A	365	279	24.2	–11.4	–9.4
			D→O_{Ox}	284	193	18.4	–17.4	–16.4

S3.1.4 Loading 3 H_2O/Mg^{2+} – Site ABC

A symmetric structure containing 18 water molecules per unit cell (3 H_2O/Mg^{2+}) is obtained starting from the experimental structure[6] with 5 H_2O/Mg^{2+} removing the water molecules at sites D and E. The hydrogen atoms are added to the water oxygens and to the linker to maximize the number of H-bonds. For this motif, the water at site A donates two H-bonds along the pore direction. A first H-bond is formed with water at site B similarly to loading 2 H_2O/Mg^{2+} sites AB. A second one is donated by water at site A to water at site B. In addition, water at site B donates to consecutive water at site C. Hence, creating a complete chain of H-bonds along the pore direction(–C–A–B–) at each corner of the hexagonal pore. Moreover, water at site C donates an H-bond to a water molecule at site B belonging to a consecutive chain connecting each chain in the perpendicular direction with respect to the pore direction, as shown in Figure S3.4. All the H-bonds, measures and orientations for this structure can be seen in Figure S3.5. All the distances and angles values of the H-bonds for loading 3 H_2O/Mg^{2+} of Mg-MOF-74 for each water at different sites are reported in Table S3.2.

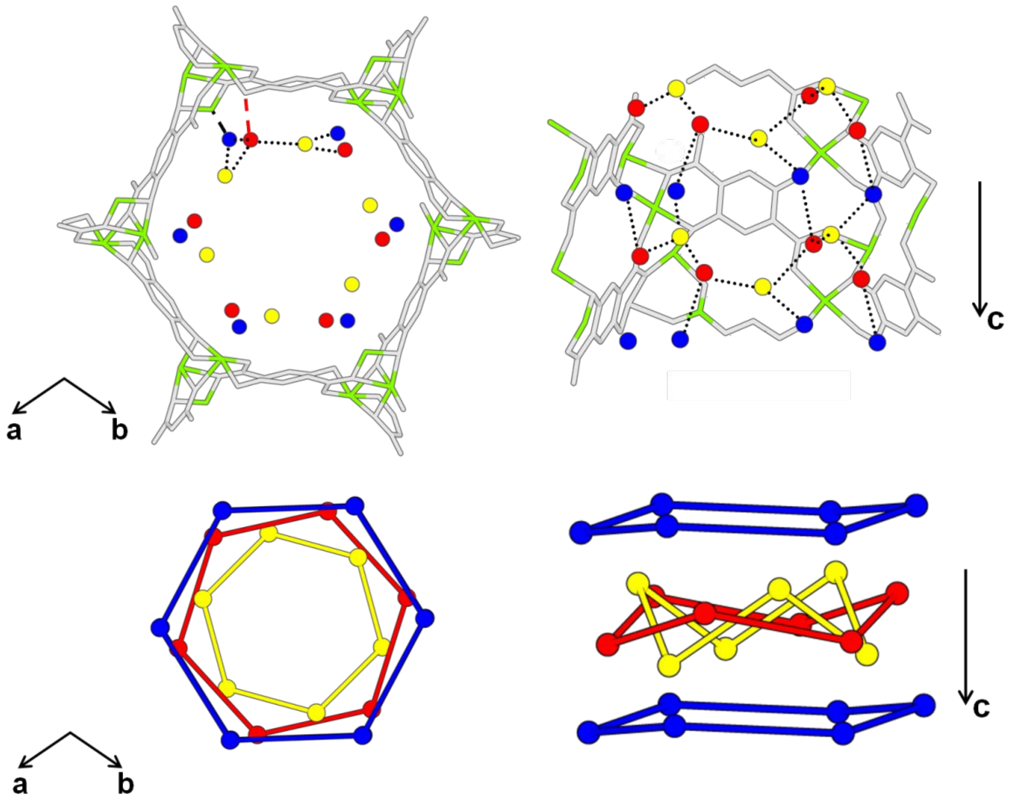

Figure S3.4. Top: Two views of the DFT-optimized structure of $Mg_2(dobdc)\cdot(H_2O)_6$. **Bottom**: Arrangement of water molecules at site A, B and C. Color code: carbon and oxygen of $(dobdc)^{4-}$ linker – gray; magnesium – light green; water adsorbed at site A (Mg^{2+}) – blue; at site B – red; and at site C – yellow. Symmetry equivalent sites are connected by lines for visual guidance. The lines do not indicate the H-bond network.

Table S3.2. Details of H-bonds for loading n = 3 H_2O/Mg^{2+}: number, donor and acceptor sites, distances R [pm] and O—H—O (α) angle [°] as well as pair interaction energy, ΔE [kJ/mol] at PBE+D3 and CCSD(T) level.

site	Color	Number (don, acc)	H-bond	R(O···O)	R(H···O)	α	ΔE	
							PBE+D3	CCSD(T)
A (Mg^{2+})	blue	2 (2, 0)	A→B	320	225	164.2	–18.5	–15.8
			A→C	287	193	158.6	–19.3	–17.3
B	red	4 (2, 2)	B→C	298	200	172.2	–24.3	–20.9
			B→O_{Ph}	287	188	171.2	–14.7	–14.6
C	yellow	3 (1, 2)	C→B	275	175	171.0	–19.9	–16.5

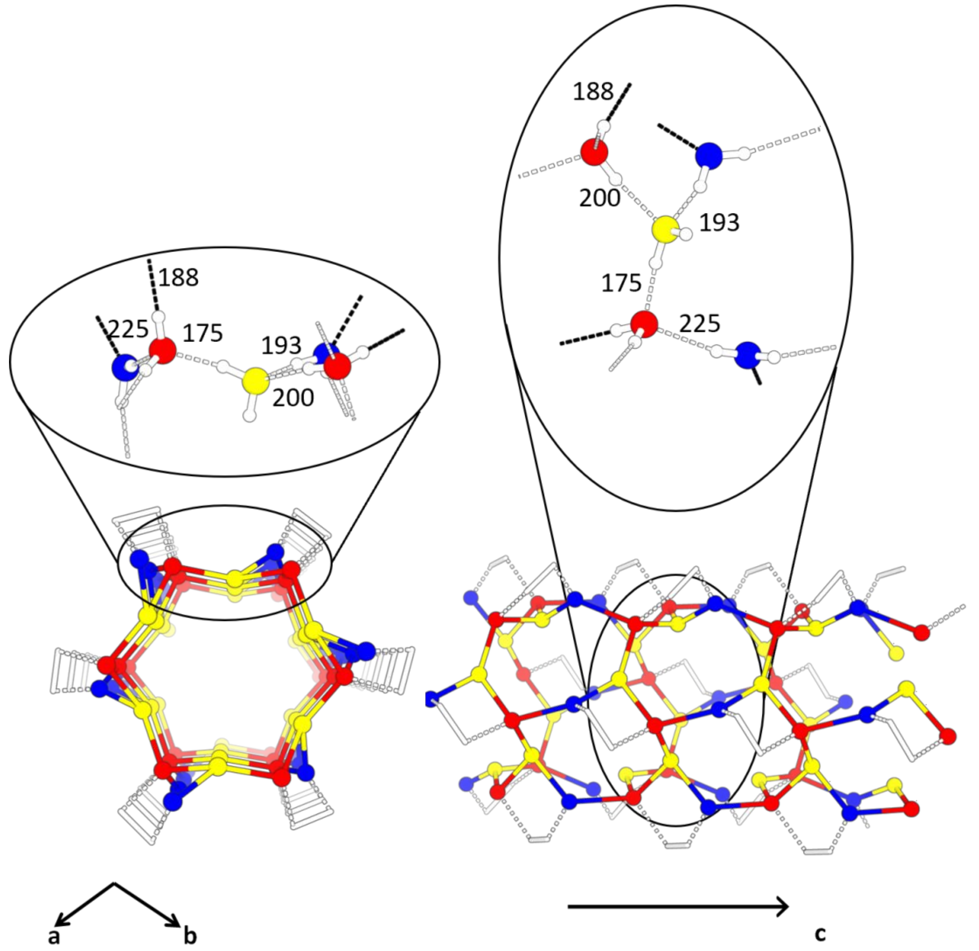

Figure S3.5. Two views of the DFT-optimized structure of $Mg_2(dobdc)\cdot(H_2O)_6$ and cluster of water showing details of interactions of water molecule at site A, B, C and the MOF. Distances in pm. Color code: $(dobdc)^{4-}$ linker and magnesium – white; water adsorbed at site A (Mg^{2+}) – blue; at site B – red; and at site C – yellow.

S3.1.5 Loading 4 H_2O/Mg^{2+} – Site ABCD

In the case of loading 4 H_2O/Mg^{2+} four distinct sites are occupied: A, B, C, and D, represented by the colors blue, red, yellow, and green, respectively, in Figure S3.6. Hence, with the loading of 4 H_2O/Mg^{2+}, the monolayer on the surface reaches completion. This configuration has a network of H-bonds illustrated in Figure S3.6 and Figure S3.7. Along the perpendicular direction to the pore, two chains of regular H-bonds emerge. The first chain is formed between the water at site B and the water molecules at site C (red and yellow, respectively), as represented in Fig S8 similar to what observed for loading 3 H_2O/Mg^{2+}. Both waters participate in reciprocal H-bonding of similar lengths (177 and 188 pm). Another one forms between the water at site A (blue) and the water at site D (forest green) (–A–D–A–D–). Here, the water at Mg^{2+} establishes a strong H-bond with the water adsorbed at site D (175 pm), which subsequently forms a weaker bond with the succeeding water at the Mg^{2+} site (220 pm). Additionally, an H-bond parallel to the pore direction is formed between the water at site A (Mg^{2+}) and the water at site B, similar to the configurations observed in the loading 2 H_2O/Mg^{2+} (AB) and 3 H_2O/Mg^{2+} (ABC). Furthermore, an H-bond is donated by the water at site D to the water at site C. Finally, two additional H-bonds are donated by water at sites B and C to the phenolic and acid oxygens of the framework, as observed in the loading 2 H_2O/Mg^{2+} configuration sites AB and AD (Section S3.1.6). Details of H-bond values and orientations can be found in Table S3.3.

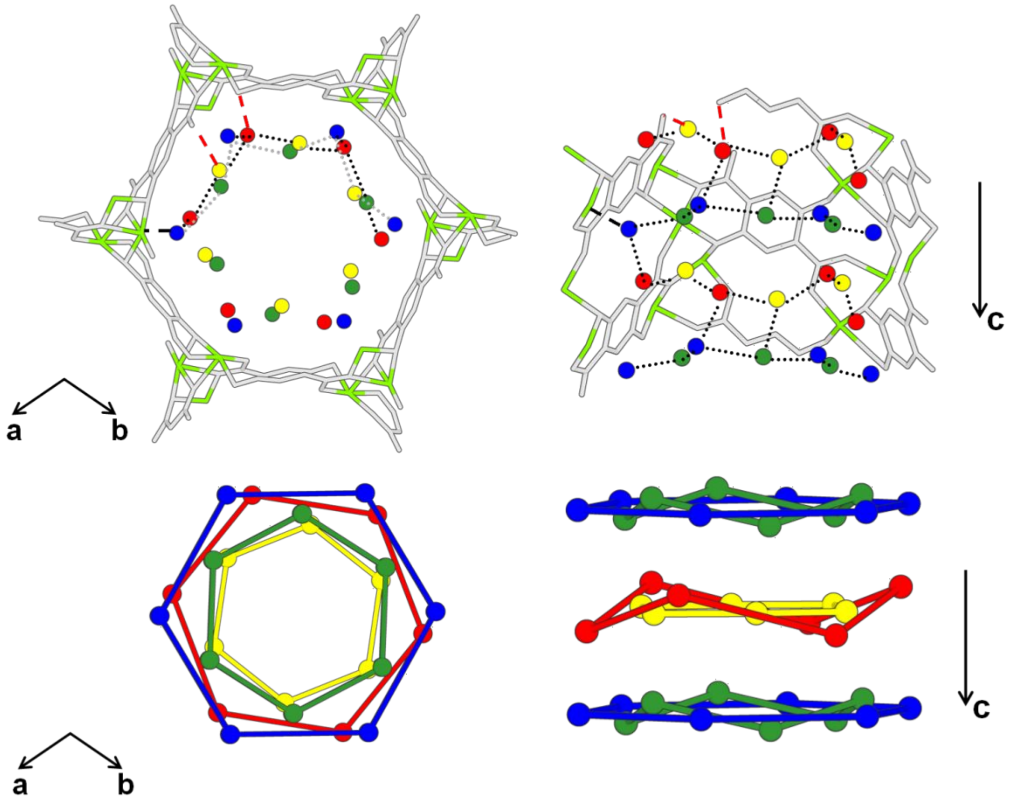

Figure S3.6. Top: Two views of the DFT-optimized structure of $Mg_2(dobdc)\cdot(H_2O)_8$. **Bottom**: Arrangement of water molecules at site A, B, C and D. Color code: carbon and oxygen of $(dobdc)^{4-}$ linker – gray; magnesium – light green; water adsorbed at site A (Mg^{2+}) – blue; at site B – red; at site C – yellow; and at site D – forest green. Symmetry equivalent sites are connected by lines for visual guidance. The lines do not indicate the H-bond network.

Table S3.3. Details of H-bonds for loading n = 4 H_2O/Mg^{2+}: number, donor and acceptor sites, distances R [pm] and O–H–O (α) angle [°] as well as pair interaction energy, ΔE [kJ/mol] at PBE+D3 and CCSD(T) level.

site	Color	Number (don, acc)	H-bond	R(O···O)	R(H···O)	α	ΔE	
							PBE+D3	CCSD(T)
A (Mg^{2+})	blue	3 (2, 1)	A→B	285	188	163.1	–17.4	–14.4
			A→D	264	164	169.4	–15.5	–11.5
B	red	4 (2, 2)	B→C	273	175	164.5	–19.7	–16.3
			B→O_{Ph}	285	186	176.1	–14.2	–14.1
C	green	4 (2, 2)	C→B	273	177	160.3	–21.6	–18.5
			C→O_{Ox}	274	179	161.0	–15.2	–13.9
D	yellow	3 (2, 1)	D→A	334	243	154.7	–18.7	–16.6
			D→C	276	184	155.0	–17.5	–15.4

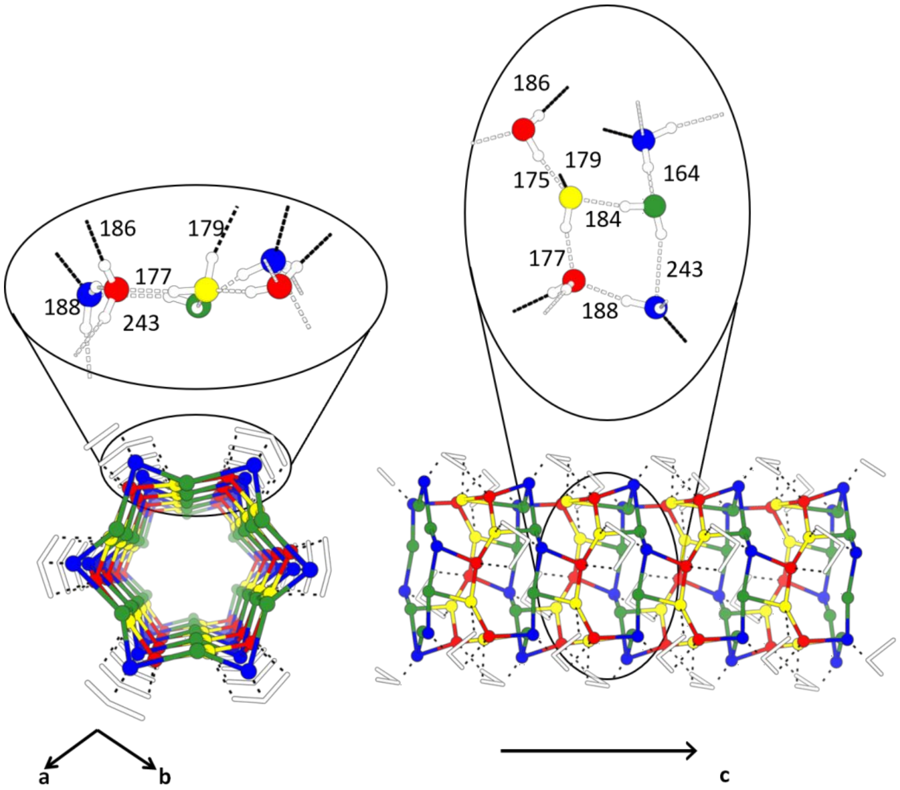

Figure S3.7. Two views of DFT-optimized structure of $Mg_2(dobdc)\cdot(H_2O)_8$ and cluster of water showing details of interactions of water at site A, B, C, D and the MOF. Distances in pm. Color code: $(dobdc)^{4-}$ linker and magnesium – white; water adsorbed at site A (Mg^{2+}) – blue; at site B – red; at site C – yellow; and at site D – forest green.

S3.1.6 Loading 5 H_2O/Mg^{2+} – site ABCDE

The fifth and final water molecule is absorbed on top of the monolayer within the pore center, completely filling the remaining pore space. This symmetrical structure, including 30 water molecules per unit cell (5 H_2O/Mg^{2+}), is obtained by adding hydrogen atoms to water oxygens and to the (dobdc)$^{4-}$ linkers into the experimental structure,[6] followed by optimization at the DFT level (space group $R\bar{3}$). This structure exhibits a network of H-bonds, for a total of 10 interactions per set of 5 water molecules per Mg^{2+}. The monolayer's H-bond pattern is similar to that observed and described for the loading of 4 H_2O/Mg^{2+}. The only notable difference is in the interaction between site C and the framework, which is absent and replaced by an additional water–water interaction between site C and E. Additionally, the second layer formed by water molecules at site E, contributes with two more H-bonds to site D, C and a consecutive water molecule at site E, forming an additional chain along the pore direction (–D–E–C–). Examining the second layer along the pore direction reveals two triangular rings of H-bonds with an O–O distance of 288 pm between three water molecules at the E sites (Figure S3.8). Distances, angles, and spatial orientation of all interactions for this motif are provided in Table S3.4 and Figure S3.9.

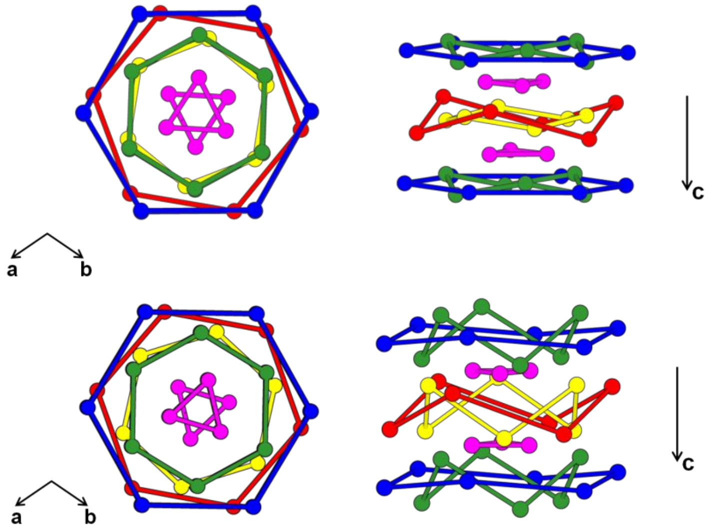

Figure S3.8. **Top**: Arrangement of water molecules from PBE+D3 for $Mg_2(dobdc)\cdot(H_2O)_{10}$. **Bottom**: Arrangement of water molecules from PXRD for $Mg_2(dobdc)\cdot(H_2O)_{10}$. Color code: water adsorbed at site A (Mg^{2+}) – blue; at site B – red; at site C – yellow; at site D – forest green; and at site E – magenta. Symmetry equivalent sites are connected by lines for visual guidance. The lines do not indicate the H-bond network.

Table S3.4. Details of H-bonds for loading n = 5 H_2O/Mg^{2+}: number, donor and acceptor sites, distances R [pm] and O–H–O (α) angle [°] as well as pair interaction energy, ΔE [kJ/mol] at PBE+D3 and CCSD(T) level.

site	Color	Number (don, acc)	H-bond	R(O···O)	R(H···O)	α	ΔE	
							PBE+D3	CCSD(T)
A (Mg^{2+})	blue	3 (2, 1)	A→B	277	179	165.8	–16.4	–13.7
			A→D	284	189	159.3	–14.5	–10.1
B	red	4 (2, 2)	B→C	276	186	160.3	–19.6	–17.3
			B→O_{Ph}	282	183	172.7	–15.1	–15.1
C	yellow	4 (2, 2)	C→B	281	186	158.3	–19.3	–15.8
			C→E	278	181	163.4	–18.9	–15.6
D	green	4 (2, 2)	D→A	311	216	164.2	–19.5	–16.0
			D→C	275	176	171.2	–17.2	–14.7
E	magenta	4 (2, 2)	E→D	282	184	170.7	–21.4	–18.6
			E→E	288	199	149.7	–18.1	–13.4

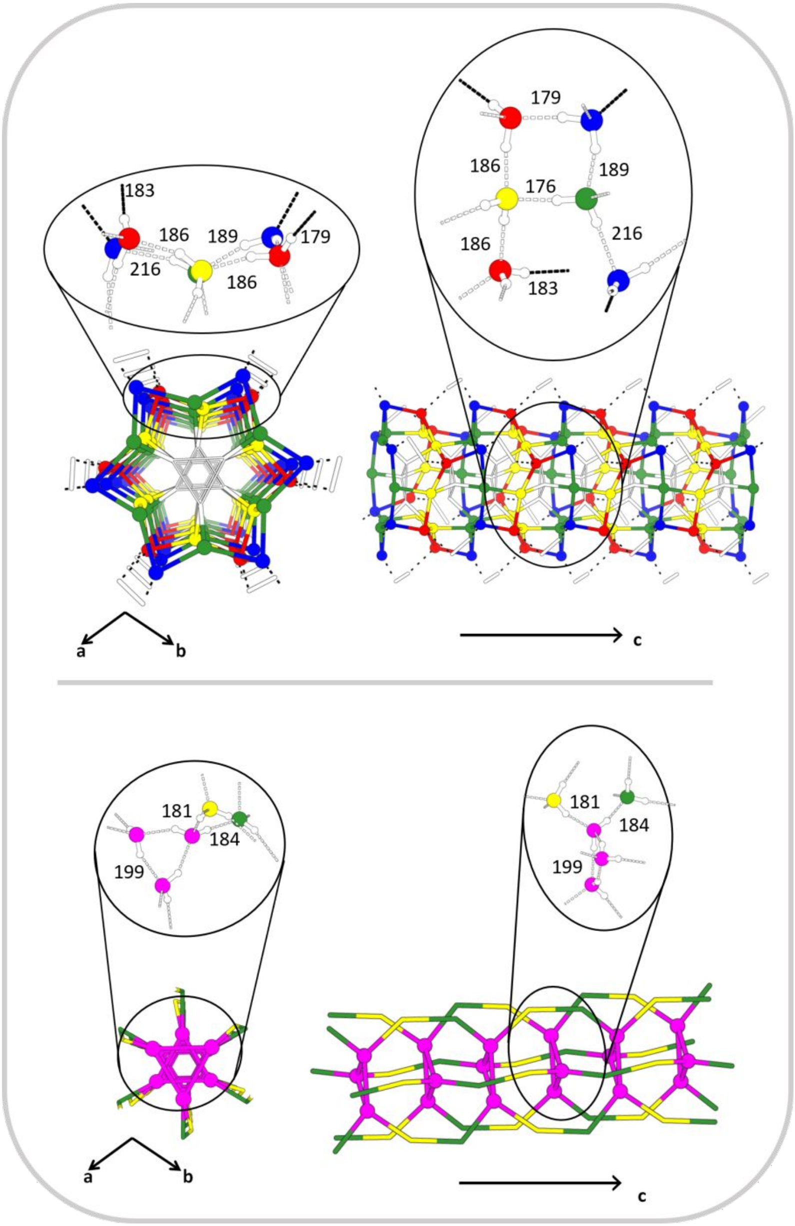

Figure S3.9. Top panel: Arrangement of water molecules from the DFT-optimized structure of $Mg_2(dobdc)\cdot(H_2O)_{10}$ monolayer, and cluster of water showing details of interactions of water at site A, B, C, D, E and the MOF. **Bottom panel**: Arrangement of water molecules from the DFT-optimized structure of $Mg_2(dobdc)\cdot(H_2O)_{10}$ second layer of water, and cluster of water showing details of interactions of water at sites C, D, E. Distances in pm. Color code: $(dobdc)^{4-}$ linker and magnesium – white; water adsorbed at site A (Mg^{2+}) – blue; at site B – red; at site C – yellow; at site D – forest green; and at site E – magenta.

S3.2 Additional Structures

S3.2.1 Loading 0–1 H_2O/Mg^{2+}

Two loadings (0.167 H_2O/Mg^{2+} and loading 0.5 H_2O/Mg^{2+}) are considered which exhibit only partially occupied Mg^{2+} sites. For loading 0.167 H_2O/Mg^{2+}, only one water molecule is adsorbed at one of the 6 available Mg^{2+} sites. For loading 0.5 H_2O/Mg^{2+}, a symmetric structure is created where 3 water molecules in the unit cell are adsorbed at three non-adjacent Mg^{2+} sites. For loading 1 (6 water molecules per unit cell), a symmetric structure in which all Mg^{2+} sites are occupied with one water molecule is obtained. Moreover, some further structures for loadings 0.5 and 1 are investigated. A structure where 3 water molecules create a water trimer (loading 0.5) and 2 further structures for loading 1 H_2O/Mg^{2+}: a three dimers structure and a two trimers structure. However, none of these structures is chosen to simulate the adsorption of water on Mg-MOF-74 as there is no advantage in terms of adsorption electronic energy compared to the symmetric structures. Figure S3.11 provides a pore view of the adsorption motif details, while Table S4.5 presents the corresponding thermodynamic values at 298 K for reference.

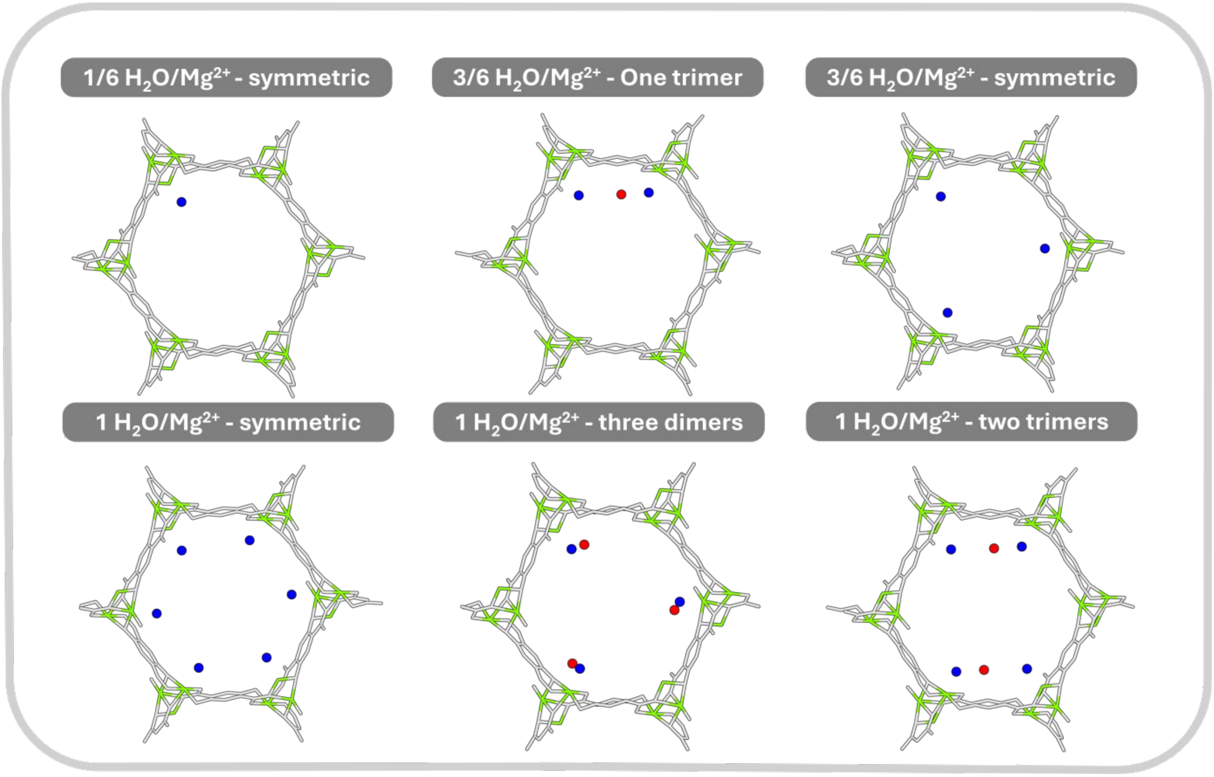

Figure S3.11. Views of the DFT-optimized structures for loadings of 1/6 H_2O per Mg^{2+}, a trimer configuration and a symmetric configuration for a loading of 3/6 (= 0.5) H_2O per Mg^{2+}, as well as symmetric, three-dimer, and two-trimer configurations for a loading of 6/6 (= 1) H_2O per Mg^{2+}. Color code: carbon and oxygen atoms of the $(dobdc)^{4-}$ linker in gray; magnesium in light green; water adsorbed at site A (Mg^{2+}) in blue; and the second water molecule forming a hydrogen bond to the first one in forest green.

S3.2.2 Summary of Additional Structures

We have broadly sampled the potential energy surface for the different loadings to ensure that the most stable adsorption motifs were identified. Table S3.6 summarizes the structures considered, some of which have already been discussed above. The adsorption motifs A, AB, ABC, ABCD, and ABCDE, which are used for the isotherm predictions in all but one case (loading 4 H_2O/Mg^{2+}), are already the most stable configurations at the PBE+D3 level and remain so in all cases after applying the high-level correction. This confirms our approach of starting from the experimentally determined O atom positions available for loading 5 H_2O/Mg^{2+} and subsequently removing water molecules.

Table S3.6. Adsorption energies per water molecule, Δ*E*, and enthalpies, Δ*H* (298 K), for all located stationary points for different loadings, in kJ/mol. In addition, the space group, SG, is specified and, if any, the imaginary wavenumbers, ν (i cm^{-1}), are given. The structures are labeled as in the manuscript. A prime (′) denotes structures with similar oxygen atom positions but different hydrogen-bond connectivity. Structures shown in bold are used for the adsorption isotherm calculations.

H_2O/Mg^{2+}	Structures	SG	Δ*E*	ΔH	ν cm^{-1}
1/6	One monomer	$P1$	–81.2	–76.0	–
1/2	Three monomers	$R3$	–78.9	–72.8	–
1/2	One trimer	$P1$	–76.8	–70.5	–
**1**	**A** (six monomers)	$\boldsymbol{R\bar{3}}$	**–80.1**	**–74.2**	–
1	Three dimers	$R3$	–76.1	–69.5	–
1	Two trimers	$P\bar{1}$	–75.4	–69.9	–
**2**	**AB**	$\boldsymbol{R\bar{3}}$	**–78.2**	**–71.6**	–
2	Simulated annealing	$P1$	–78.4	–71.4	–
2	AD	$R\bar{3}$	–75.8	–69.3	–
**3**	**ABC**	$\boldsymbol{R\bar{3}}$	**–76.6**	**–69.7**	–
3	ABD	$P1$	−71.3	–	28
3	AB(C/D)[a]		−72.2	–	71
3	AB(C/D[a]		−74.0	–	19; 42; 53
3	ACD	$P1$	–60.1	–74.1	–
**4**	**ABCD**	$\boldsymbol{R\bar{3}}$	**–76.6**	**–69.5**	–
4	ABCD’	$R\bar{3}$	−75.5	–	27
**5**	**ABCDE**	$\boldsymbol{R\bar{3}}$	**–79.6**	**–72.0**	–
5	ABCDE’	$R\bar{3}$	−74.7	–	26; 51; 56; 71; 77
5	ABCDE”	$R\bar{3}$	−72.3	–	52; 53; 58; 65; 81

[a] The third water molecule is located in between sites C and D.

For loading 1 H_2O/Mg^{2+}, the additional hydrogen bonds formed in the three-dimer and two-trimer structures do not compensate for the loss of direct interactions between the water molecules and the Mg^{2+} ions. For loading 2 H_2O/Mg^{2+}, the **AD** motif is another possible configuration but is less stable, as discussed above. We also performed simulated annealing to obtain a third configuration, which features more hydrogen bonds but is overall less strongly bound than **AB**. For loading 3 H_2O/Mg^{2+}, we considered the motifs **ABC**, **ABD**, and **ACD**. Both **ABD** and **ACD** are significantly less stable than **ABC**. We also identified structures in which the water molecules are located between sites **C** and **D**. These configurations are less stable than **ABC** and exhibit imaginary frequencies, indicating that they are not true minima but rather shallow saddle points. For loading 4 H_2O/Mg^{2+}, the first layer covering the internal MOF surface is filled, leaving only the pore center available. This restricts the possible arrangements of the water molecules. We identified two distinct hydrogen-bond networks, **ABCD** and **ABCD'**, and used the more stable one, **ABCD**, which is also the only configuration for which no imaginary frequencies were found. Similarly, for loading 5 H_2O/Mg^{2+}, the highest loading, the pore space is fully occupied, which strongly restricts the possible arrangements of the water molecules. While the O atoms remain in the experimentally observed positions, we explored several possible hydrogen-bond networks and selected the most stable one, **ABCDE**. This is also the only configuration for which no imaginary frequencies were found, making it the only true minimum identified at this loading.

S4. ADSORPTION ENERGIES AND THERMODYNAMICS

S4.1. Thermodynamic Quantities per Water Molecule

This section presents the thermodynamic quantities related to water adsorption on Mg-MOF-74, including adsorption energies, enthalpies, entropies, and Gibbs free energies. These values are reported as averages per water molecule to enable a direct comparison of the stability of different adsorption motifs at various loadings, see Table S4.1.

Table S4.1. Thermodynamic contributions to the adsorption of *n* H_2O per Mg^{2+} ion in Mg-MOF-74, $Mg_2(dobdc)\cdot(H_2O)_{2n}$, normalized per water molecule, (Equation 3 in the main text). Electronic energies, ΔE, zero-point vibrational energies, ΔE_{ZPV}, thermal energy correction, ΔE_{therm}, enthalpies, ΔH, entropy contributions, $-T\Delta S$, where ΔS is the entropy of adsorption and T is the temperature, and Gibbs free energies, ΔG, are included. Vibrational contributions are calculated using the harmonic oscillator rigid rotor approximation at the PBE+D3 level for T = 298 K and standard pressure (0.1 MPa). The coupled cluster (CCSD(T)) corrections, ΔCC, defined in (Equation 1 in the main text), are calculated using the PBE+D3 adsorption structures. The number of H-bonds per water molecule, *m*, is also given.

n	Sites	PBE+D3						*m*	CCSD(T)	
		ΔE	ΔE_{ZPV}	ΔE_{therm}	ΔH	$-T\Delta S$	ΔG		ΔCC	ΔG+ΔCC
1	A	−80.1	7.9	0.5	−74.2	40.7	−33.5	–	0.3	−33.5
2	AB	−78.3	10.0	−0.9	−71.6	43.9	−27.7	1.5	1.5	−26.2
3	ABC	−76.6	10.5	−1.1	−69.7	44.1	−25.6	3.0	3.8	−21.8
4	ABCD	−76.7	11.5	−1.8	−69.5	45.9	−23.7	3.5	4.8	−18.9
5	ABCDE	−79.6	12.2	−2.1	−72.0	46.7	−25.2	3.8	5.9	−19.3

The PBE+D3 adsorption energy, is strongest for the adsorption of the first water molecule at site A. As additional water molecules are adsorbed, the adsorption weakens, plateauing at 3 H_2O/Mg^{2+}. The adsorption energy for the fourth water molecule remains similar, whereas the adsorption energy of the fifth molecule, which completes pore filling, gets stronger again.

The zero-point vibrational energy contributions, ΔE_{ZPV}, induce a destabilization of the adsorption energies by 7.9 to 12.2 kJ/mol per water molecule. However, thermal energy contributions, ΔE_{therm}, combined with RT, partially offset this destabilization. As a result, the adsorption enthalpies differ from the adsorption energies by only 0.5 and –2.1 kJ/mol per water molecule.

As more water molecules are adsorbed, the entropic destabilization increases. The entropic contribution, $-T\Delta S$, rises from 40.9 kJ/mol for a loading of 1 H_2O/Mg^{2+} to 46.7 kJ/mol for 5 H_2O/Mg^{2+}. This trend deviates from the commonly observed enthalpy-entropy compensation, where stronger adsorption typically correlates with a greater reduction in degrees of freedom, leading to more significant entropic destabilization. In our case, the exception arises from the

behavior of the first adsorbed water molecule, which retains a degree of rotational freedom along the Mg–O axis. The addition of a second water molecule forms a water dimer, restricting this rotational motion and resulting in a substantial increase in entropy from 40.9 to 43.9 kJ/mol. Subsequent adsorption steps show smaller changes in entropy, yet completing the water monolayer with the fourth molecule and fully filling the pore with the fifth molecule yield the highest entropic destabilizations. This may be attributed to the formation of more rigid, fully connected hydrogen-bond networks.

As the number of adsorbed water molecules increases, the total number of H-bonds rises from 2 at a loading of 2 H_2O/Mg^{2+} to 10 at 5 H_2O/Mg^{2+}, corresponding to an increase from 1.0 to 2.0 H-bonds per water molecule. This increase in H-bonds correlates with the increase in the high-level correction, which ranges from 1.5 kJ/mol per water molecule at 2 H_2O/Mg^{2+} to 5.9 kJ/mol per water molecule at 5 H_2O/Mg^{2+}. This trend can be attributed to the systematic overestimation of H-bonds by the PBE+D3 GGA functional. As the number of H-bonds per water molecule increases, so does the magnitude of the high-level correction, reflecting the cumulative effect of these overestimations.

The high-level corrected Gibbs free energy follows the same decreasing trend as the PBE+D3 Gibbs free energy, plateauing at a loading of 3 H_2O/Mg^{2+}. However, the loss in exergonicity per water molecule with increasing loading is more pronounced in the high-level corrected values, as the destabilizing effect of the high-level corrections becomes more significant at higher loadings. Consequently, while the PBE+D3 adsorption energies for the first and fifth water molecules are nearly identical, the much larger high-level correction for the fifth adsorption distinguishes it from the first. This results in the first adsorption being much stronger, as reflected in the high-level corrected Gibbs free energies.

S4.2 Adsorption Energies at 313 K

Table S4.2. Electronic energies of adsorption (ΔE), zero-point vibrational energies (ΔE_{ZPV}), enthalpies (ΔH), entropic contributions ($-T\Delta S$) where T is the temperature and S is the entropy of adsorption and Gibbs free energies of adsorption ΔG. All thermodynamic functions are obtained within the harmonic approximation PBE+D3 level for every loading at T = 298 and 313 K. The pressure is P = 0.1 MPa.

n	Sites			298 K			313 K		
H_2O/Mg^{2+}		ΔE	ΔE_{ZPV}	ΔH	$-T\Delta S$	ΔG	ΔH	$-T\Delta S$	ΔG
1	A	–80.1	7.9	–74.2	–40.7	–33.5	–74.1	–42.4	–31.7
2	AB	–78.3	10.0	–71.6	–43.9	–27.7	–71.6	–46.0	–25.5
3	ABC	–76.6	10.5	–69.7	–44.1	–25.6	–69.6	–46.2	–23.4
4	ABCD	–76.7	11.5	–69.5	–45.9	–23.7	–69.5	–48.1	–21.4
5	ABCDE	–79.6	12.2	–72.0	–46.7	–25.2	–72.0	–49.1	–22.9

Table S4.3. Electronic energies of adsorption (ΔE), zero-point vibrational energies (ΔE_{ZPV}), Gibbs free energies of adsorption ΔG, enthalpies (ΔH) and entropic contributions ($-T\Delta S$) where T is the temperature and S is the entropy of adsorption for the first, the second, the third, the fourth and the fifth layer. All thermodynamic functions are obtained within the harmonic approximation at the PBE+D3 level for every loading at T = 298 and 313 K. The pressure is P = 0.1 MPa

			298 K			313 K		
Sites	ΔE	ΔE_{ZPV}	ΔH	$-T\Delta S$	ΔG	ΔH	$-T\Delta S$	ΔG
Bare MOF→A	–80.1	7.9	–74.2	–40.7	–33.5	–74.1	–42.4	–31.7
A→AB	–76.5	12.1	–69.0	–47.1	–22.0	–69.0	–49.6	–19.4
AB→ABC	–73.2	11.4	–65.7	–44.4	–21.3	–65.7	–46.5	–19.2
ABC→ABCD	–77.1	14.6	–69.1	–51.2	–17.9	–69.2	–54.0	–15.2
ABCD→ABCDE	–91.0	14.9	–81.7	–50.2	–31.5	–81.8	–52.8	–29.0

Table S4.4. Electronic energies of adsorption (ΔE), CCSD(T) High-level correction for H-bonds interactions (ΔCC), Gibbs free energies of adsorption ΔG, for each adsorption step. All data is obtained at T = 298 and 313 K. The pressure is P = 0.1 MPa.

	PBE+D3	CCSD(T)	298 K		313 K	
Sites	ΔE	ΔCC	ΔG	ΔG+ΔCC	ΔG	ΔG+ΔCC
0→A	–80.1	0.3	–33.5	–33.2	–31.7	–31.4
A→AB	–76.5	3.0	–22.0	–19.0	–19.4	–16.4
A→AD	–71.5	6.6	–20.2	–13.6	–17.7	–11.1
AB→ABC	–73.2	8.4	–21.3	–12.9	–19.2	–10.8
ABCD→ABCDE	–91.0	10.2	–31.5	–21.3	–29.0	–18.8

S4.3 Loading 0–1 H_2O/Mg^{2+}

PBE+D3 results for the loading of 0 to 1 H_2O/Mg^{2+} are presented in Table S4.5. Analysis of the adsorption electronic energy leads to the conclusion that when certain metal sites are already occupied, additional water molecules preferentially adsorb at the remaining available metal sites rather than the linker sites.

Table S4.5. Results obtained at PBE+D3 level. Electronic energies of adsorption (Δ*E*), zero-point vibrational energies (ΔE_{ZPV}), Gibbs free energies of adsorption (Δ*G*), enthalpies (Δ*H*) and entropic contributions (–*T*Δ*S*) where *T* is the temperature and *S* is the entropy of adsorption for loading *n* = 0 to 1 H_2O/Mg^{2+}. All thermodynamic functions are obtained within the harmonic approximation PBE+D3 level for every loading at *T* = 298 K. The pressure is *P* = 0.1 MPa.

n	Symm.	PBE+D3		298 K		
H_2O/Mg^{2+}		Δ*E*	ΔE_{ZPV}	Δ*G*	Δ*H*	−*T*Δ*S*
$^1/_6$	Symmetric	–81.2	5.7	–41.4	–76.0	34.6
$^1/_2$	Symmetric	–78.9	7.3	–35.7	–72.8	37.1
$^1/_2$	One trimer	–76.8	9.0	–27.9	–70.5	42.6
1	Symmetric	–80.2	8.1	–33.6	–74.2	40.6
1	Three dimers	–76.1	9.9	–25.6	–69.5	43.9
1	Two trimers	–75.4	8.1	–28.7	–69.9	41.2

S4.4 Comparison Between Space Group P1 and $R\bar{3}$ PBE+D3 Results

Table S4.6. PBE+D3 adsorption energies per water (Δ*E*) and zero-point vibrational energies (ΔE_{ZPV}). All energies in kJ/mol: comparing optimized motifs with ($R\bar{3}$) and without symmetry reinforcement (*P*1).

n	Sites	$R\bar{3}$		*P*1	
H_2O/Mg^{2+}		Δ*E*	ΔE_{ZPV}	Δ*E*	ΔE_{ZPV}
1	A	–80.1	7.9	–80.2	8.1
2	AB	–78.3	10.0	–78.3	10.0
2	AD	–75.8	9.7	–75.6	9.8
3	ABC	–76.6	10.5	–76.6	10.6
4	ABCD	–76.7	11.5	–76.7	11.3
5	ABCDE	–79.6	12.2	–76.7	11.2

S5. ADSORPTION ISOTHERMS AND ISOBARS

S5.1 Deviation Between *Multi-site Langmuir* Predictions and Experiments

To evaluate the accuracy of our calculated Gibbs free energies for each step of water loading, $\Delta G_{\mathrm{n-1 \to n}}$, we compare simulated adsorption isotherms to experimental data. We simulated isotherms using our calculated CCSD(T)-quality free energies and varied each $\Delta G_{\mathrm{n-1 \to n}}$ by ±2 kJ/mol. The resulting uncertainty intervals contain all experimental isotherm data points, suggesting that our Gibbs free energies are accurate to within approximately ±2 kJ/mol compared to the experiments by Ahn[41] and Walton.[42]

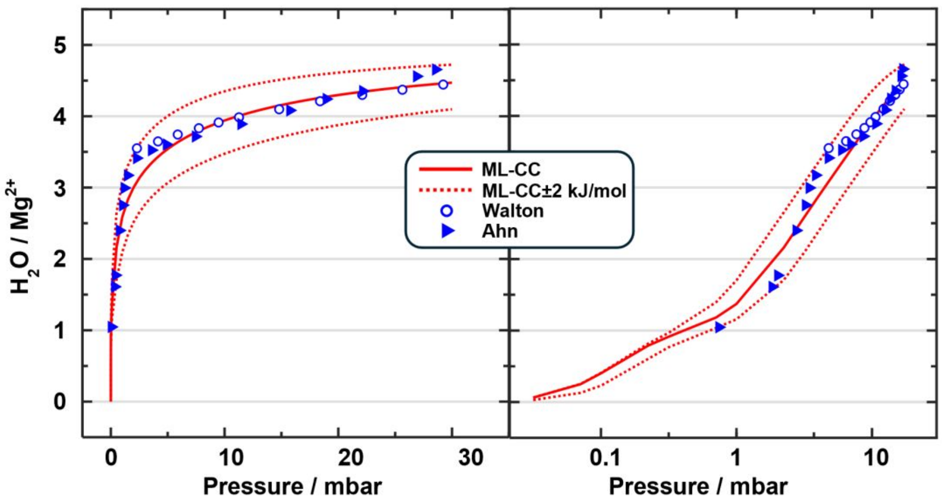

Figure S5.1. Left: *Multi-site Langmuir* (ML) with inclusion of CCSD(T) corrections (CC, red), Red dotted lines represent the ±2 kJ/mol, Experimental isotherms (light blue symbols) taken from Walton[42] (blue circles) and Ahn[41] (blue triangles) for 298 K. **Right:** the logarithmic scale of the pressure.

S5.2. *Multi-step Langmuir* Model

In addition to the *Multi-site Langmuir* model described in section 3.3, we have used a model which explicitly considers the sequential nature of adsorption. The addition of a water depends on the population of the preceding site:

$$0 + H_2O \xrightarrow{K_1} A \tag{S5.1a}$$

$$0 + 2\,H_2O \xrightarrow{K_1} A + H_2O \xrightarrow{K_2} AB \tag{S5.1b}$$

$$0 + 3\,H_2O \xrightarrow{K_1} A + 2\,H_2O \xrightarrow{K_2} AB + H_2O \xrightarrow{K_3} ABC \tag{S5.1c}$$

....

The equilibrium adsorption constant for loading *n* is defined as:

$$K_n = exp\left(-\frac{\Delta G_{\mathrm{n-1 \to n}}}{\mathrm{R}T}\right). \tag{S5.2}$$

The coverage for each of the states with *n* water molecules at pressure *P,* with consideration of previous adsorption steps is given by the following Langmuir expressions:

$$\theta_1 = \frac{K_1 P}{1+ K_1 P} \tag{S5.3a}$$

$$\theta_2 = \frac{K_1 K_2 P^2}{1+ K_1 P + K_1 K_2 P^2} \tag{S5.3b}$$

$$\dots$$

$$\theta_n = \frac{K_1 \dots K_n P^n}{1+ \sum_{i=1}^{n} K_1 \dots K_n P^i} \tag{S5.3c}$$

The total coverage at a given pressure and temperature is the sum of the coverages of all five steps

$$\theta_{\mathrm{total}} = \sum_{n=1}^{5} \theta_n \tag{S5.4}$$

For both adsorption models, Figure S5.2 shows the individual contributions of the five adsorption states to the total isotherm using the CCSD(T)-corrected Gibbs free energies from Table 2. The two models differ only for the filling of states ABCD and ABCDE, but only marginally. A sites are filled already at 0.1 mbar and at 10 mbar also B sites are fully occupied.

The *Multi-site Langmuir* model shows a slightly better agreement with experiment, and it is also the simpler model. Together, this is why we report the *Multi-site Langmuir* results in the main text.

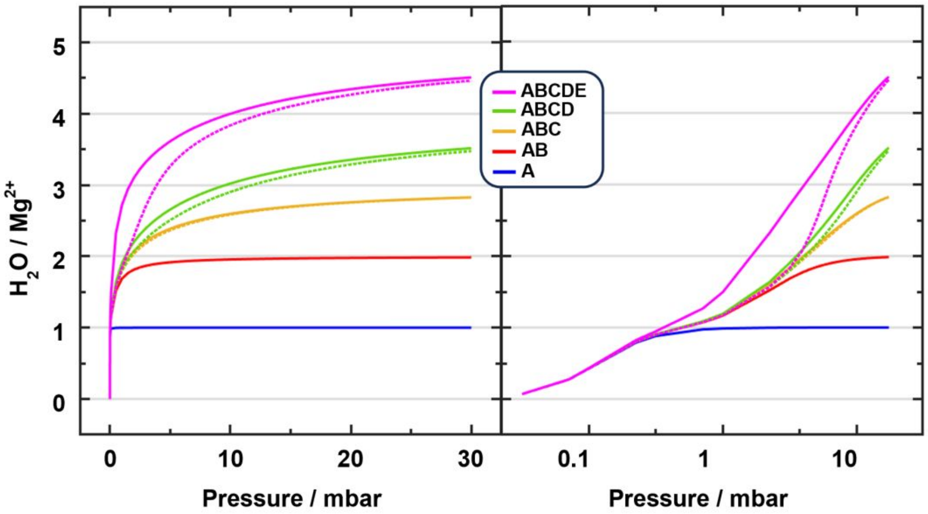

Figure S5.2. Left: Individual contributions of each step for the two isotherm models: *Multi-site Langmuir* (solid-line) and *Step-wise Langmuir* (dotted-line). Color code: site A – blue; site AB – red; site ABC – yellow; site ABCD – green; and site ABCDE – magenta. Isotherm obtained with CCSD(T) Gibbs free energies per step, reported in Table 2. **Right**: logarithmic pressure scale.

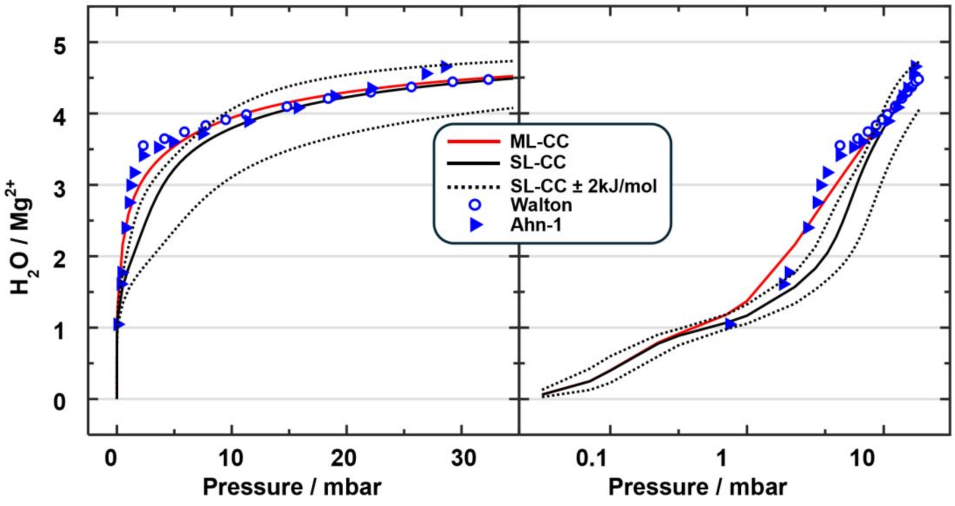

Figure S5.3. **Left:** Predicted adsorption isotherms compared to experiment (298 K). *Multi-step Langmuir* model (SL-CC, black line), compared to *Multi-site Langmuir* model (ML-CC, red line) based on CCSD(T) corrections. Black dotted lines represent the ±2 kJ/mol. Experimental isotherms (light blue symbols) taken from Walton[42] (triangles) and Ahn[41] (squares). **Right:** the logarithmic scale of the pressure.

S5.3 Scaling and Preoccupation Effects on Isotherm Simulations

We employed two distinct methods to account for defects and pre-occupation of metal sites before measurements. The first involves applying a global scaling factor to the adsorption isotherms, with values ranging from 1.0 for an ideal sample to 0.6 to model the regime of variations in the experimentally observed isotherms depicted in the left panels of Figure S5.4. Reducing the scaling factor can be interpreted as a uniform reduction in water uptake while maintaining the original Langmuir model's shape. To understand the effect of incomplete evacuation, the right panels of Figure S5.4 model six additional scenarios in which 1 to 6 of the six A sites are assumed to be preoccupied by water and thus inactive for adsorption of a further water molecule at site A. In this case, a more pronounced impact on water uptake is observed at lower pressures compared to higher pressures. Overall, across the entire pressure range, its effect is less drastic compared to the scaling factor. As a result, both scaling and assuming preoccupation with water effectively downscale the isotherms. The difference in the two methods lies in their behavior across distinct pressure regimes. In the case of scaling, the influence is relatively modest at low pressures, gradually amplifying as the pressure increases. On the contrary, assuming preoccupation, the effect is more pronounced at lower pressures and diminishes gradually as the pressure increases.

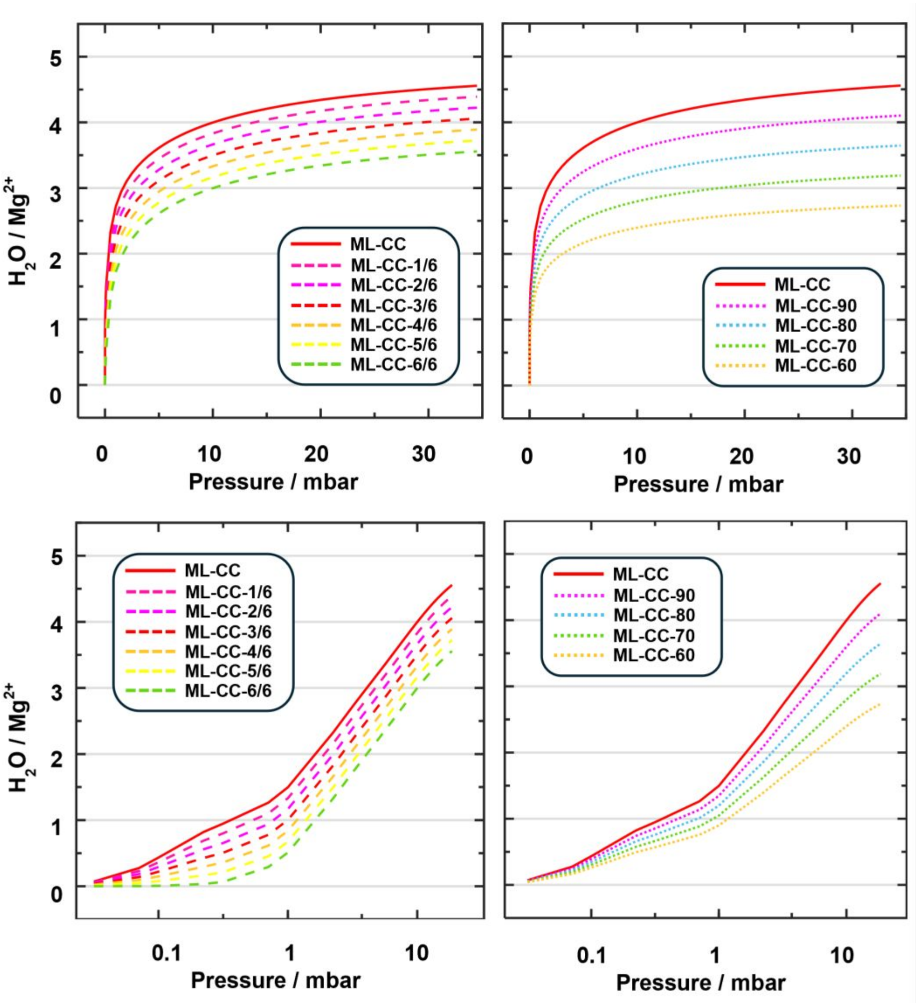

Figure S5.4. Top left: Isotherms obtained considering 1/6, 1/3, 1/2, 2/3, 5/6, and all A sites of the *Multi-site Langmuir* local-harmonic ML-CC isotherm are not available for adsorption (dashed line – light red, magenta, red, orange, yellow, and green respectively). **Top right**: Isotherms obtained introducing a scaling factor of 0.9, 0.8, 0.7, 0.6 (dotted line – red, magenta, light*; Multi-site Langmuir* blue, green, and yellow respectively) on ML-CC isotherm. **Bottom panels:** Logarithmic scale of the pressure.

S5.4 Scaling Experimental Isotherms

Here, we compare two isotherm measurements conducted at 298 K by Yaghi and coworkers (2014)[43] and Ahn and coworkers (2012).[41]

Although the Ahn isotherm reaches higher maximum loadings than Yaghi's, the shapes of the isotherms align remarkably well. This alignment becomes more evident when Ahn‘s isotherm is proportionally scaled down by a factor of 0.86 to align with Yaghi's isotherm.

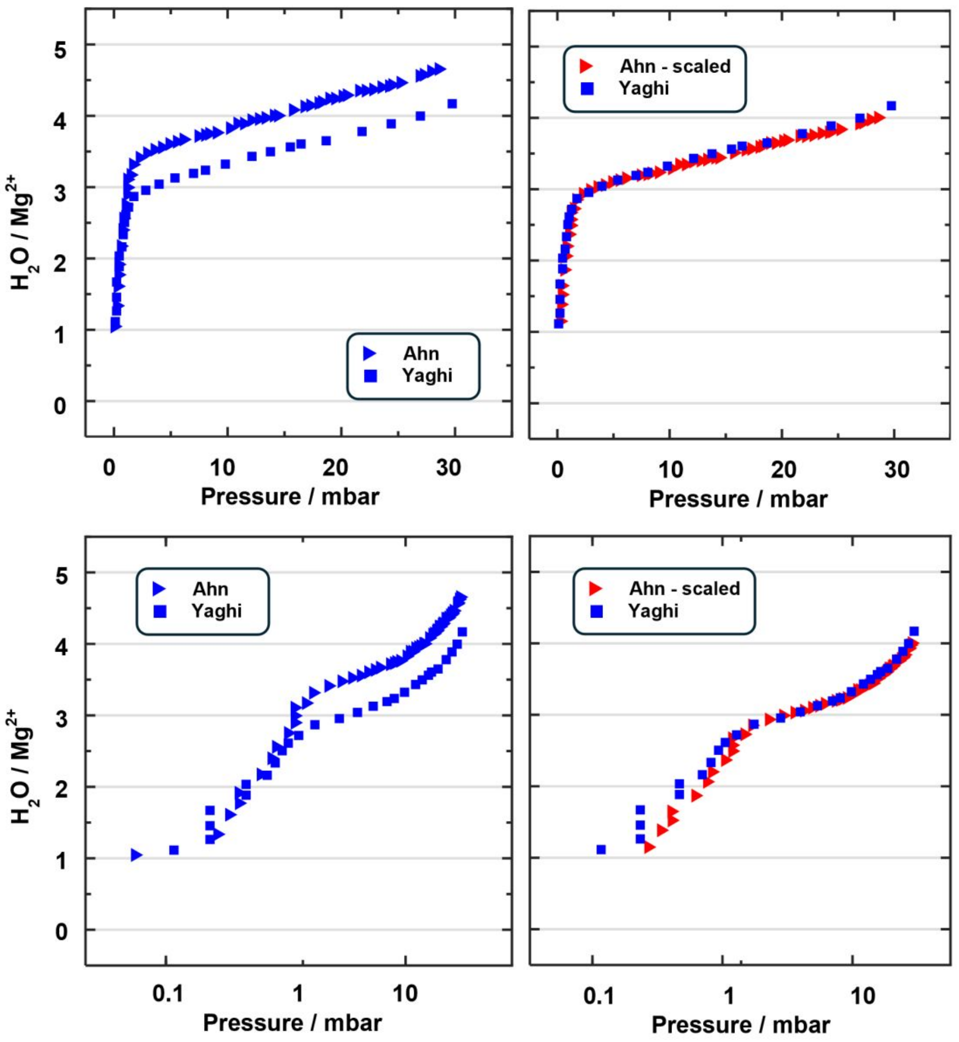

Figure S5.5. Top left: Experimental Isotherms of Ahn (2012) and Yaghi (2014). **Top right:** Scaled (factor = 0.86) Ahn isotherm and non-scaled Yaghi isotherm. **Bottom panels:** Logarithmic scale of the pressure.

S5.5 Comparison with Monte Carlo Simulations and a DFT-Derived Force-Field

The first-principles Monte Carlo (FPMC) simulations by Siepmann and co-workers, using PBE+D3 energies,[44] predict significantly lower water loadings at a given pressure, as well as lower maximum uptake, compared to our Multi-site Langmuir isotherm model which is also based on PBE+D3. For example, at 20 mbar, MC/PBE+D3 underestimates the loading by approximately 1 H_2O/Mg^{2+} while our Multi-site Langmuir model overestimates it by about 0.75 H_2O/Mg^{2+} (see Figure S5.6). MC simulations become increasingly expensive at higher water loadings (water densities), as the likelihood of successfully "transfer[ing water molecules] between the vapor and adsorbent phases"[44] drops sharply. Therefore, the discrepancies in predicted loading and saturation may stem from insufficient convergence of the FPMC simulations.

The DFT-derived force field (FF) developed by Smit and co-workers offers a strong compromise between accuracy and computational efficiency. It reproduces the general shape of our PBE+D3 based isotherm while achieving better agreement with the experimental isotherms. However, it shows the same limitations as our PBE+D3 Multi-site Langmuir model, including an overestimation of water uptake and an inability to capture the step-like adsorption behavior only observed with the more accurate CCSD(T) results. A direct comparison with our PBE+D3 results is not possible, as the underlying methodologies differ; Smit's FF is based on the vdW-DF2[45] functional which may explain the better agreement with experiments in the low-loading regime, and they employ grand-canonical Monte Carlo (GCMC).

Similar to the FPMC simulations by Siepmann and co-workers, GCMC simulations by Smit and co-workers do not reach the experimentally observed maximum water loading. This limitation may arise from the same challenge: GCMC simulations become increasingly expensive at higher loadings, as the likelihood of successful water insertion drops steeply. That said, the deviation from experiment is much smaller for Smit's GCMC simulations than for the FPMC simulations, likely because force field-based simulations can be converged more thoroughly. An additional source of error at high loadings may lie in the parameterization of the FF itself. As noted by the authors, "our force field was parametrized to DFT binding energies for one H_2O molecule in the framework at a time and thus should be more accurate at lower concentrations of H_2O where $H_2O{-}H_2O$ interactions do not play a dominant role".[46]

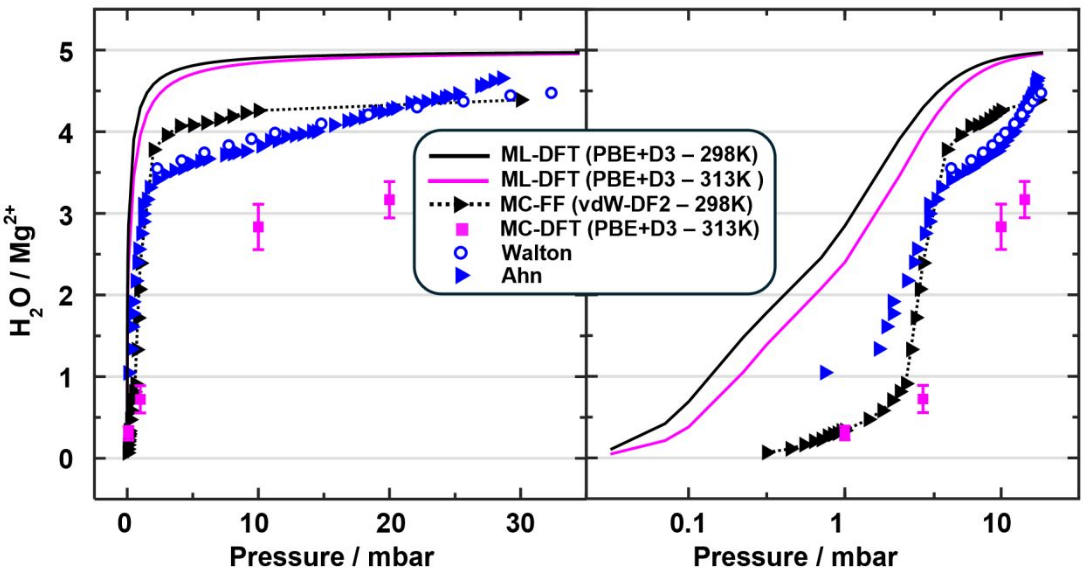

Figure S5.6. *Left: Multi-site Langmuir* isotherms calculated with PBE+D3 for 298 K (ML-DFT, black) and 313 K (ML-DFT, magenta) compared to a 298 K isotherm obtained with a vdW-DF2 force field by Smit and co-workers[46] (MC-FF, black) and to Monte Carlo results obtained with PBE+D3 by Siepmann and coworkers[44] for an *N*, *p*, *T* ensemble at 313 K (MC-DFT, magenta). Experimental isotherms (298 K, blue symbols) taken from Walton[42] (circles) and Ahn[41] (triangles). *Right:* Logarithmic pressure scale.

S5.6 Comparison Between *Multi-site*, *Multi-step,* and *Two-step Langmuir* Desorption Isobars

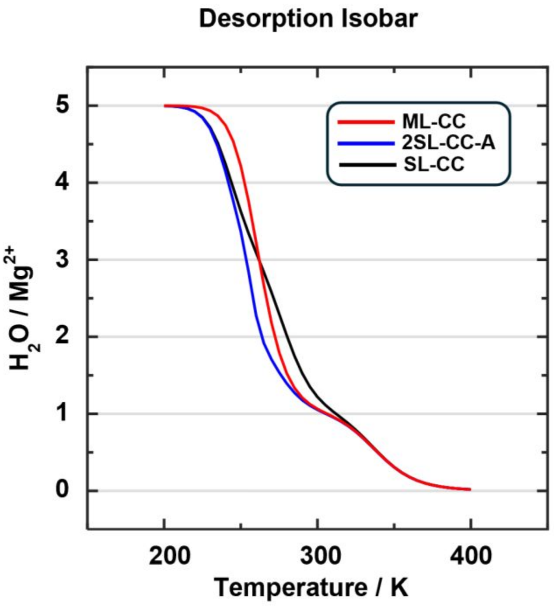

Figure S5.7 Predicted isobar (5 Pa). *Multi-site-Langmuir* (ML) with inclusion of CCSD(T) corrections (CC, red), *Multi-step Langmuir* (SL) model based on the same results (CC, black) and a *Two-step* (2SL) *Langmuir* (5 H_2O/MOF - 1 H_2O/MOF and 1 H_2O/MOF - 0 H_2O/MOF) at CCSD(T) level (CC, blue).

S6. REFERENCES

1. Queen, W. L.; Brown, C. M.; Britt, D. K.; Zajdel, P.; Hudson, M. R.; Yaghi, O. M., Site-Specific CO_2 Adsorption and Zero Thermal Expansion in an Anisotropic Pore Network. *J. Phys. Chem. C* **2011,** *115*, 24915-24919, https://doi.org/10.1021/jp208529p.

2. Zhou, W.; Wu, H.; Yildirim, T., Enhanced H_2 Adsorption in Isostructural Metal−Organic Frameworks with Open Metal Sites: Strong Dependence of the Binding Strength on Metal Ions. *J. Am. Chem. Soc.* **2008,** *130*, 15268-15269, https://doi.org/10.1021/ja807023q.

3. Wu, H.; Simmons, J. M.; Srinivas, G.; Zhou, W.; Yildirim, T., Adsorption Sites and Binding Nature of CO_2 in Prototypical Metal−Organic Frameworks: A Combined Neutron Diffraction and First-Principles Study. *J. Phys. Chem. Lett.* **2010,** *1*, 1946-1951, https://doi.org/10.1021/jz100558r.

4. Caskey, S. R.; Wong-Foy, A. G.; Matzger, A. J., Dramatic Tuning of Carbon Dioxide Uptake via Metal Substitution in a Coordination Polymer with Cylindrical Pores. *J. Am. Chem. Soc.* **2008,** *130*, 10870-10871, https://doi.org/10.1021/ja8036096.

5. Xiao, D.; Bloch, E.; Mason, J.; Queen, W.; Hudson, M.; Planas, N.; Borycz, J.; Dzubak, A.; Verma, P.; Lee, K., , J. Yano, S. Bordiga, DG Truhlar, L. Gagliardi, CM Brown and JR Long. *Nat. Chem* **2014,** *6*, 590-595.

6. Dietzel, P. D. C.; Blom, R.; Fjellvåg, H., Base-Induced Formation of Two Magnesium Metal-Organic Framework Compounds with a Bifunctional Tetratopic Ligand. *Eur. J. Inorg. Chem.* **2008,** *2008*, 3624-3632, https://doi.org/10.1002/ejic.200701284.

7. Sillar, K.; Hofmann, A.; Sauer, J., Ab Initio Study of Hydrogen Adsorption in MOF-5. *J. Am. Chem. Soc.* **2009,** *131*, 4143-4150, https://doi.org/10.1021/ja8099079.

8. Sillar, K.; Sauer, J., Ab Initio Prediction of Adsorption Isotherms for Small Molecules in Metal–Organic Frameworks: The Effect of Lateral Interactions for Methane/CPO-27-Mg. *J. Am. Chem. Soc.* **2012,** *134*, 18354-18365, https://doi.org/10.1021/ja307076t.

9. Alessio, M.; Bischoff, F. A.; Sauer, J., Chemically accurate adsorption energies for methane and ethane monolayers on the MgO(001) surface. *Phys. Chem. Chem. Phys.* **2018,** *20*, 9760-9769, https://doi.org/10.1039/C7CP08083B.

10. Bischoff, F. A.; Alessio, M.; Berger, F.; John, M.; Rybicki, M.; Sauer, J. *Multi-Level Energy Landscapes: The MonaLisa Program*, Humboldt-University: Berlin: www.chemie.hu-berlin.de/de/forschung/quantenchemie/monalisa/, 2019.

11. Nosé, S., A unified formulation of the constant temperature molecular dynamics methods. *J. Chem. Phys.* **1984,** *81*, 511-519, https://doi.org/10.1063/1.447334.

12. Hoover, W. G., Canonical dynamics: Equilibrium phase-space distributions. *Physical Review A* **1985,** *31*, 1695-1697, https://doi.org/10.1103/PhysRevA.31.1695.

13. Borycz, J.; Paier, J.; Verma, P.; Darago, L. E.; Xiao, D. J.; Truhlar, D. G.; Long, J. R.; Gagliardi, L., Structural and Electronic Effects on the Properties of Fe_2(dobdc) upon Oxidation with N_2O. *Inorg. Chem.* **2016,** *55*, 4924-4934, https://doi.org/10.1021/acs.inorgchem.6b00467.

14. Krivy, I.; Gruber, B., A unified algorithm for determining the reduced (Niggli) cell. *Acta Cryst. A* **1976,** *32*, 297-298, https://doi.org/10.1107/S0567739476000636.

15. Křivý, I.; Gruber, B., A unified algorithm for determining the reduced (Niggli) cell. *Acta Cryst. A* **1976,** *32*, 297-298, https://doi.org/10.1107/S0567739476000636.

16. Kundu, A.; Piccini, G.; Sillar, K.; Sauer, J., Ab Initio Prediction of Adsorption Isotherms for Small Molecules in Metal–Organic Frameworks. *J. Am. Chem. Soc.* **2016,** *138*, 14047-14056, https://doi.org/10.1021/jacs.6b08646.

17. Riplinger, C.; Neese, F., An efficient and near linear scaling pair natural orbital based local coupled cluster method. *J. Chem. Phys.* **2013,** *138*, https://doi.org/10.1063/1.4773581.

18. Neese, F., The ORCA program system. *Wiley Interdiscip. Rev. Comput. Mol. Sci.* **2012,** *2*, 73-78, https://doi.org/https://doi.org/10.1002/wcms.81.

19. Neese, F., Software update: the ORCA program system, version 4.0. *Wiley Interdiscip. Rev. Comput. Mol. Sci.* **2018,** *8*, e1327, https://doi.org/https://doi.org/10.1002/wcms.1327.

20. Boys, S. F.; Bernardi, F., The calculation of small molecular interactions by the differences of separate total energies. Some procedures with reduced errors. *Mol. Phys.* **1970,** *19*, 553-566, https://doi.org/10.1080/00268977000101561.

21. Jensen, F., Estimating the Hartree—Fock limit from finite basis set calculations. *Theor. Chem. Acc.* **2005,** *113*, 267-273, https://doi.org/10.1007/s00214-005-0635-2.

22. Helgaker, T.; Klopper, W.; Koch, H.; Noga, J., Basis-set convergence of correlated calculations on water. *J. Chem. Phys.* **1997,** *106*, 9639-9646, https://doi.org/10.1063/1.473863.

23. Woon, D. E.; Dunning, T. H., Jr., Gaussian basis sets for use in correlated molecular calculations. III. The atoms aluminum through argon. *J. Chem. Phys.* **1993,** *98*, 1358-1371, https://doi.org/10.1063/1.464303.

24. Kendall, R. A.; Dunning, T. H., Jr.; Harrison, R. J., Electron affinities of the first-row atoms revisited. Systematic basis sets and wave functions. *J. Chem. Phys.* **1992,** *96*, 6796-6806, https://doi.org/10.1063/1.462569.

25. Weigend, F.; Häser, M.; Patzelt, H.; Ahlrichs, R., RI-MP2: optimized auxiliary basis sets and demonstration of efficiency. *Chem. Phys. Lett.* **1998,** *294*, 143-152, https://doi.org/10.1016/S0009-2614(98)00862-8.

26. Manna, D.; Kesharwani, M. K.; Sylvetsky, N.; Martin, J. M. L., Conventional and Explicitly Correlated ab Initio Benchmark Study on Water Clusters: Revision of the BEGDB and WATER27 Data Sets. *J. Chem. Theory Comput.* **2017,** *13*, 3136-3152, https://doi.org/10.1021/acs.jctc.6b01046.

27. Windeck, H.; Berger, F.; Sauer, J., Chemically accurate predictions for water adsorption on Brønsted sites of zeolite H-MFI. *Phys. Chem. Chem. Phys.* **2024,** *26*, 23588-23599, https://doi.org/10.1039/D4CP02851A.

28. Curtiss, L.; Frurip, D.; Blander, M., Studies of molecular association in H_2O and D_2O vapors by measurement of thermal conductivity. *J. Chem. Phys.* **1979,** *71*, 2703-2711, https://doi.org/10.1063/1.438628.

29. Feyereisen, M. W.; Feller, D.; Dixon, D. A., Hydrogen Bond Energy of the Water Dimer. *J. Phys. Chem.* **1996,** *100*, 2993-2997, https://doi.org/10.1021/jp952860l.

30. Halkier, A.; Koch, H.; Jørgensen, P.; Christiansen, O.; Nielsen, I. M. B.; Helgaker, T., A systematic ab initio study of the water dimer in hierarchies of basis sets and correlation models. *Theor. Chem. Acc.* **1997,** *97*, 150-157, https://doi.org/10.1007/s002140050248.

31. Kuhs, W. F.; Lehmann, M. S., The structure of the ice Ih by neutron diffraction. *J. Phys. Chem.* **1983,** *87*, 4312-4313, https://doi.org/10.1021/j100244a063.

32. Krukau, A. V.; Vydrov, O. A.; Izmaylov, A. F.; Scuseria, G. E., Influence of the exchange screening parameter on the performance of screened hybrid functionals. *J. Chem. Phys.* **2006,** *125*, 224106, https://doi.org/10.1063/1.2404663.

33. Perdew, J. P.; Ernzerhof, M.; Burke, K., Rationale for mixing exact exchange with density functional approximations. *J. Chem. Phys.* **1996,** *105*, 9982-9985, https://doi.org/10.1063/1.472933.

34. Ernzerhof, M.; Scuseria, G. E., Assessment of the Perdew–Burke–Ernzerhof exchange-correlation functional. *J. Chem. Phys.* **1999,** *110*, 5029-5036, https://doi.org/10.1063/1.478401.

35. Adamo, C.; Barone, V., Toward reliable density functional methods without adjustable parameters: The PBE0 model. *J. Chem. Phys.* **1999,** *110*, 6158-6170, https://doi.org/10.1063/1.478522.

36. Becke, A. D., Density-functional thermochemistry. III. The role of exact exchange. *J. Chem. Phys.* **1993,** *98*, 5648-5652.

37. Lee, C.; Yang, W.; Parr, R. G., Development of the Colle-Salvetti correlation-energy formula into a functional of the electron density. *Phys. Rev. B* **1988,** *37*, 785.

38. Stephens, P. J.; Devlin, F. J.; Chabalowski, C. F.; Frisch, M. J., Ab Initio Calculation of Vibrational Absorption and Circular Dichroism Spectra Using Density Functional Force Fields. *J. Phys. Chem.* **1994,** *98*, 11623-11627, https://doi.org/10.1021/j100096a001.

39. Kresse, G.; Furthmüller, J., Efficient iterative schemes for ab initio total-energy calculations using a plane-wave basis set. *Phys. Rev. B* **1996,** *54*, 11169-11186, https://doi.org/10.1103/PhysRevB.54.11169.

40. Kresse, G.; Furthmüller, J., Efficiency of ab-initio total energy calculations for metals and semiconductors using a plane-wave basis set. *Comput. Mater. Sci.* **1996,** *6*, 15-50, https://doi.org/https://doi.org/10.1016/0927-0256(96)00008-0.

41. Yang, D.-A.; Cho, H.-Y.; Kim, J.; Yang, S.-T.; Ahn, W.-S., CO_2 capture and conversion using Mg-MOF-74 prepared by a sonochemical method. *Energy Environ. Sci.* **2012,** *5*, 6465-6473, https://doi.org/10.1039/C1EE02234B.

42. Schoenecker, P. M.; Carson, C. G.; Jasuja, H.; Flemming, C. J. J.; Walton, K. S., Effect of Water Adsorption on Retention of Structure and Surface Area of Metal–Organic Frameworks. *Ind. Eng. Chem. Res.* **2012,** *51*, 6513-6519, https://doi.org/10.1021/ie202325p.

43. Furukawa, H.; Gándara, F.; Zhang, Y.-B.; Jiang, J.; Queen, W. L.; Hudson, M. R.; Yaghi, O. M., Water Adsorption in Porous Metal–Organic Frameworks and Related Materials. *J. Am. Chem. Soc.* **2014,** *136*, 4369-4381, https://doi.org/10.1021/ja500330a.

44. Fetisov, E. O.; Shah, M. S.; Long, J. R.; Tsapatsis, M.; Siepmann, J. I., First principles Monte Carlo simulations of unary and binary adsorption: CO_2, N_2, and H_2O in Mg-MOF-74. *Chem. Commun.* **2018,** *54*, 10816-10819, https://doi.org/10.1039/C8CC06178E.

45. Lee, K.; Murray, É. D.; Kong, L.; Lundqvist, B. I.; Langreth, D. C., Higher-accuracy van der Waals density functional. *Phys. Rev. B* **2010,** *82*, 081101, https://doi.org/10.1103/PhysRevB.82.081101.

46. Mercado, R.; Vlaisavljevich, B.; Lin, L.-C.; Lee, K.; Lee, Y.; Mason, J. A.; Xiao, D. J.; Gonzalez, M. I.; Kapelewski, M. T.; Neaton, J. B.; Smit, B., Force Field Development from Periodic Density Functional Theory Calculations for Gas Separation Applications Using Metal–Organic Frameworks. *J. Phys. Chem. C* **2016,** *120*, 12590-12604, https://doi.org/10.1021/acs.jpcc.6b03393.
